# *In vivo* regulation of bacterial Rho-dependent transcription termination by the nascent RNA

**DOI:** 10.1016/j.jbc.2022.102001

**Published:** 2022-04-29

**Authors:** Passong Immanual R. Chhakchhuak, Ranjan Sen

**Affiliations:** 1 Laboratory of Transcription, Center for DNA Fingerprinting and Diagnostics, Uppal, Hyderabad, India; 2 Graduate Studies, Regional Center for Biotechnology, Faridabad, Haryana, India

**Keywords:** rho, *rut* sites, transcription termination, qRT-PCR, NusG, descriptors, EC, elongation complex, MG, malachite green, NTP, nucleotide triphosphate, NusG-CTD, NusG interacting with C-terminal domain, PBS, primary binding site, RNAP, RNA polymerase, RP, reverse primer, TSS, transcription start site

## Abstract

Bacterial Rho is a RNA-dependent ATPase that functions in the termination of transcription. The *in vivo* nature of the bacterial Rho-dependent terminators, as well as the mechanism of the Rho-dependent termination process, are not fully understood. Here, we measured the *in vivo* termination efficiencies of 72 Rho-dependent terminators in *Escherichia coli* by systematically performing qRT-PCR analyses of cDNA prepared from mid-log phase bacterial cultures. We found that these terminators exhibited a wide range of efficiencies, and many behaved differently *in vivo* compared to the predicted or experimentally determined efficiencies *in vitro*. Rho-utilization sites (*rut* sites) present in the RNA terminator sequences are characterized by the presence of C-rich/G-poor sequences or C > G bubbles. We found that weaker terminators exhibited a robust correlation with the properties (size, length, density, etc.) of these C > G bubbles of their respective *rut* sites, while stronger terminators lack this correlation, suggesting a limited role of *rut* sequences in controlling *in vivo* termination efficiencies. We also found that *in vivo* termination efficiencies are dependent on the rates of ATP hydrolysis as well as Rho-translocation on the nascent RNA. We demonstrate that weaker terminators, in addition to having *rut* sites with diminished C > G bubble sizes, are dependent on the Rho-auxiliary factor, NusG, *in vivo*. From these results, we concluded that *in vivo* Rho-dependent termination follows a nascent RNA-dependent pathway, where Rho-translocation along the RNA is essential and *rut* sequences may recruit Rho *in vivo*, but Rho-*rut* binding strengths do not regulate termination efficiencies.

The bacterial transcription termination follows two pathways: intrinsic, RNA-hairpin–dependent, and extrinsic, Rho factor–dependent termination (1). Rho is a homo-hexameric protein that is capable of binding to the nascent RNA and possesses RNA-dependent ATPase function. The latter property enables it to translocate along with the nascent RNA in a 5′ to 3′ direction. The *cis*-acting Rho-binding sites on the RNA are called Rho utilization sites (*rut* sites), which are C-rich and G-poor unstructured sequences (C > G bubbles; (2)). The Rho-terminators consist of *rut* sites and downstream termination zones, where RNAs are released (transcription stop points) from the transcription elongation complex (EC) (3, 4, 5, 6). Some of the terminators may also contain auxiliary elements that allow stronger binding for Rho. These elements are proposed to include 5′-YC (Y = pyrimidine) repeats, which act as initial contact points to the N-terminal primary binding site (PBS) of Rho (5, 7, 8, 9). These terminators could be present at the beginning of the genes or within the genes (10). Recently, Nadiras *et al*. 2018 has defined the properties of the C-rich *rut* sites based on the content of %C and %G in these sequence stretches and quantitatively expressed them in terms of “descriptors”, which were used to predict the *in vitro* strength of the different Rho-dependent terminators.

The Rho-dependent termination is facilitated by the transcription elongation factor NusG (11) that directly interacts with Rho *via* its c-terminal domain (NusG-CTD) (12, 13, 14), which leads to early Rho-dependent termination *in vitro* (15, 16, 17). Among the Rho-dependent terminators, a subset of terminators (∼20%) seems to be dependent on NusG *in vivo* (10). It has been proposed that NusG accelerates the conformational changes of Rho from open to closed hexamer complex during the process of Rho-loading to RNA (18, 19).

There are two models for the mechanism of Rho-dependent termination: RNA-dependent pathway, where Rho binds to the *rut* sites on the nascent RNA and translocates along with it to catch up with the EC (6, 20, 21). In this model, the molecular motor function of Rho enables Rho to translocate along with the RNA as well as displace the RNA polymerase (RNAP) from the incoming nucleotide triphosphate (NTP) site of the EC *via* RNA-DNA hybrid shearing (22, 23, 24). More recently, an RNAP-dependent pathway model has been put forward, where Rho binds to RNAP before binding to the *rut site*. Subsequently, it is transferred to the *rut* sites of the emerging RNA as and when the latter exits out of the EC (25, 26, 27, 28). However, in both models, Rho interacts with the elongating RNAP at some point in the termination process in addition to its interactions with the nascent RNA.

Based on the *in vitro* termination efficiencies and the nature of the RNA sequences, an algorithm developed by Nadiras *et al*., 2018 predicted the genome-wide presence of various strong and weak terminators. The transcriptomic analyses had also revealed more than 1200 Rho-dependent termination units in the *Escherichia coli* genome (10). However, quantitative measurements of *in vivo* Rho-dependent termination efficiencies in different genes and *in vivo* regulation of these terminators by the nature of their cognate *rut* sites are not known. The *in vivo* Rho-dependent terminator strength, as well as the efficiency of the termination process, should be different from those obtained by *in vitro* measurements because the *in vivo* process could be affected by the mRNA-binding of ribosomes and other RNA-binding proteins, availability of NusG-CTD (NusG-CTD also binds to the ribosome) to interact with Rho, presence of different *cis*-acting elements like RNA-hairpins and riboswitches, Rho-antagonists, etc. It is also important to know how the nature and pattern of the C > G bubbles (the “descriptors”, (9)) are correlated with the *in vivo* termination efficiencies of the various Rho-dependent terminators, which in turn would decipher whether the *rut* sites regulate the *vivo*-termination process as well as the existence of the RNA-dependent pathway *in vivo*.

In this study, we have employed RT-qPCR analyses of 72 genes to identify the presence of Rho-dependent terminators and *in vivo* termination efficiencies of these terminators using *E. coli* strains expressing termination-defective Rho mutants and Rho-binding defective NusG mutants. These terminators exhibited a wide range of efficiencies and many of them behaved differently *in vivo* compared to that predicted or experimentally shown *in vitro* (9, 17). The weak or moderately strong terminators, under *in vivo* conditions, showed a positive and robust correlation with the nature (size, length, density, etc) of the C > G bubble stretches (described as “descriptors”) of the *rut* sites. This correlation disappeared as the terminator strength increased suggesting a limited role of the nature of *rut* sequences in controlling the termination efficiencies *in vivo*, even though the binding affinities of Rho for the *rut* sites were dependent on the nature of the C > G bubbles. The termination efficiencies of the majority of the terminators showed good positive correlations with their respective rates of ATPase activities of Rho on the RNA templates having the terminator-zone sequences. This indicates the Rho-translocase activity-dependence of the majority of the terminators *in vivo*. Consistent with the earlier proposals (18, 19), the *in vivo* termination efficiencies for the weak and moderately strong terminators exhibited dependence on NusG. The highly NusG-dependent terminators have significantly reduced the area, length, and density of the C > G bubbles. These data indicate the existence of nascent-RNA–dependent pathways *in vivo*, where Rho translocation along the RNA is essential for the termination.

## Results

### Measurements of *in vivo* termination efficiencies of the Rho-dependent terminators

**Figure 1:**
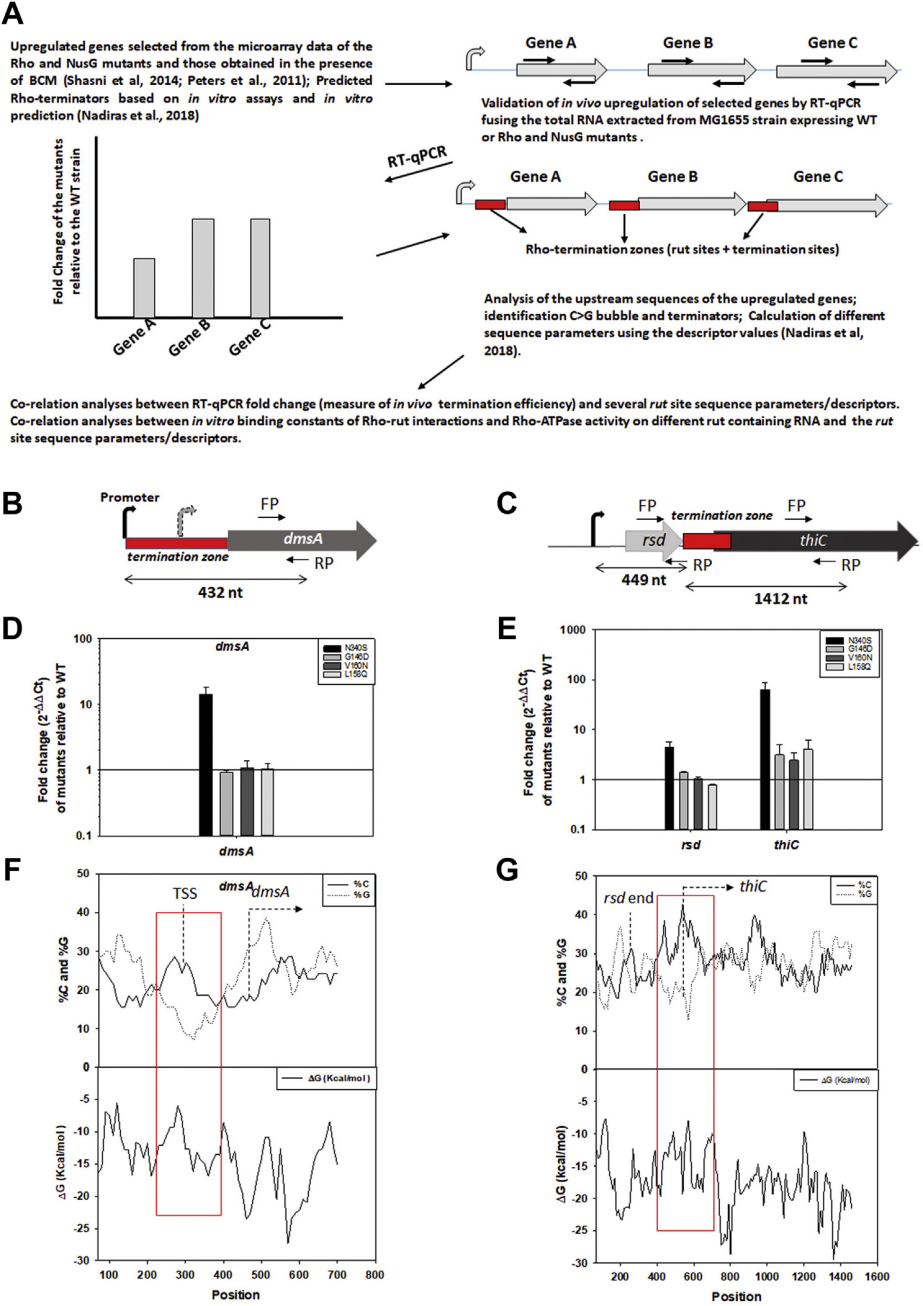
**Design of experiments.***A*, cartoon showing the methodologies used for identifying the Rho-dependent terminators and *in vivo* measurements of their termination efficiencies in *E. coli*. Fold change relative to the WT strain gives the measure of *in vivo* termination efficiencies. *Arrows* over and below the genes indicate the location of the primers used in the Rt-qPCR experiments. *Red blocks* are the identified terminators present in an operon. *B* and *C*, representative operons used in the study. FP and RP are the forward and the reverse primers used in the RT-qPCR experiments. Length of the RNA, promoter, and terminator elements are indicated. *D* and *E*, representative fold change data obtained from the RT-qPCR assays in the presence of Rho mutant, N340S, and NusG mutants, G146D, V160N, L158Q. *F* and *G*, the representative C > G bubble plots showing the %C and %G (*upper plots*) and ΔG value (*lower plots*) within the same sequence that was analyzed. Each data point is calculated from a 70 nt sliding window. FP, forward primer; RP, reverse primer.

Several genomics studies revealed the existence of genes as well as operons, expressions of which are under the control of Rho-dependent termination *in vivo* (10, 29, 30, 31). More recently, Nadiras et.al., 2018 has predicted genomic regions, which function as Rho-dependent terminators based on the dataset obtained from the *in vitro* transcription termination assays. We chose genes that are shown by the above-mentioned studies to be under the regulation of Rho-dependent termination (Fig. S1). We primarily chose the genes that are upregulated in the microarray profiles under the conditions when Rho function is compromised (detailed in Fig. S1). Later, more genes upstream or downstream of these chosen genes as well as some genes whose functional characterizations were ongoing in our laboratory were added to the primary list. We compared the level of transcribed RNA from these genes in the WT MG1655 strain relative to those obtained in the strains expressing the Rho mutant N340S and the NusG mutants, G146D, L158Q, and V160N (Fig. S2). This Rho mutant is defective in secondary RNA binding as well as severely defective in ATPase activity (17), whereas, the NusG mutants have strong binding defects for Rho (14). And hence, both the Rho and NusG mutants have defective Rho-dependent termination functions resulting in read-through transcription into the genes, which otherwise is terminated in the WT variants. This is manifested as upregulation of the expressions of the downstream genes in an RT-qPCR assay (Fig. 1*A*). Multiple primers probes were used to experimentally validate the presence of Rho-dependent termination zones upstream of these probes sites (Fig. 1, *A*–*C*). The length of the RNA transcripts of different genes that were analyzed in these assays varied from 91 to 5914 nt (Figs. 1, *B* and *C*, and S3). The enhancements of the RT-qPCR signals of each gene in the Rho or the NusG mutants relative to the WT strain give a measure of *in vivo* gene expression in those strains. The relative enhancements of gene expressions are expressed in fold-change as indicated in Figure 1, *D* and *E*. As the termination zones are present upstream of these genes (Fig. 1, *A*–*C*), the fold-change values of each gene give a semiquantitative measure of the *in vivo* termination efficiencies of the terminators present in these terminator zones. The higher fold change values indicate the higher termination efficiency and the presence of a stronger terminator.

The *rut* sites within a terminator zone are characterized by C > G sequence bubbles in the RNA sequences (sequence of non-template DNA) (2). We scanned and analyzed the sequences upstream of 72 genes that were used in the RT-qPCR assays for the presence of C > G bubbles (Fig. 1, *F* and *G*; see Experimental procedures section). We followed the methodology described by Nadiras *et al*. 2018 to define and calculate the descriptor values based on the length, size, width, etc of these bubbles (Fig. S4; definitions of each descriptor are stated in the Experimental procedures section under “*sequence selection and analyses of rut sites*” and also in the Fig. S4).

**Figure 2:**
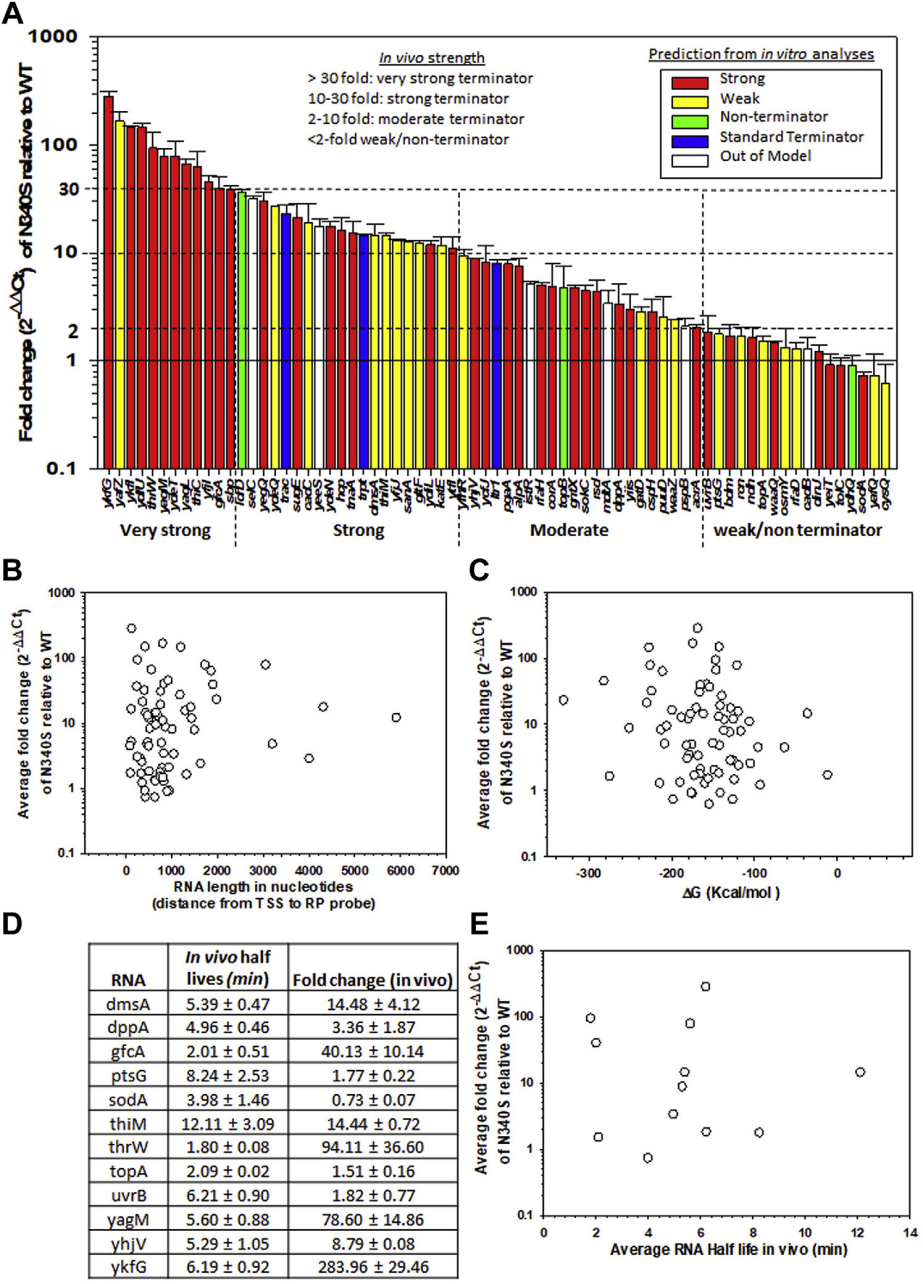
**Measurement of *in vivo* termination efficiencies**. *A*, average fold change of the gene expressions of the indicated genes in Rho N340S mutant relative to that obtained in a WT Rho strain using RT-qPCR. Error bars were obtained from the measurements of 3–4 cDNA preparations. The color codes of the bars are indicated. Termination zones were identified upstream of these indicated genes. *In vitro* predictions were from Nadiras *et al*., 2018. Different categories of terminators are indicated. *B*, plots of fold changes obtained from (*A*) against the length of RNA of each gene as indicated. TSS and RP denote the transcription start site and reverse primer, respectively (see also the cartoons in Fig. 1). *C*, plots of fold changes obtained from (*A*) against the change in folding free energy of each of the RNA transcripts measured using the Vienna RNA-folding program. *D*, Table showing the values of *in vivo* half-lives of the selected RNA expressed from the genes that were indicated in (*A*). Experimental determination of the half-lives is shown in Fig. S4. *E*, plots of these measured half-lives against the fold changes (obtained from Fig. 2*A*) of the corresponding genes. TSS, transcription start site; RP, reverse primer.

*In vitro* termination efficiencies of many Rho-dependent terminators have been measured, and an algorithm developed from these experimental data was used to predict their strength (9). However, *in vivo* termination efficiencies of these genes should be quite different as Rho-dependent termination faces many competing processes inside the cell (see Introductory paragraphs). We measured the upregulation (in terms of fold-change) of 72 genes in a strain expressing the Rho N340S mutant relative to that expressing the WT variant by RT-qPCR (Fig. 2*A*). Among them, some regions were previously identified to have Rho-dependent terminators in an *in vivo* 3′-end mapping study (31). The fold change values as well as the *in vivo* termination efficiencies varied over 2 log scales. Based on these fold-change values, we classified the terminators into the following groups: weak or nonterminator (<2 fold), moderate (2–10 fold), strong (10–30 fold), and very strong (>30) (Fig. 2*A*). Out of the 54 terminators that belong to the very strong to the moderately strong category, 31 were predicted to be strong from the *in vitro* data (9), whereas out of 17 terminators in the weak or nonterminator category, only 8 of them were predicted to be weak. Interesting to note that out of five terminators that could not be predicted properly (white bars), four were good terminators *in vivo*. This mismatch between the *in vitro* and *in vivo* might indicate that *rut* sequences *per se* do not control Rho-dependent termination *in vivo*. Since we expressed the *in vivo* termination efficiencies as fold change of the RNA expression levels relative to the WT strain, possible interferences from the presence of the multiple transcription start-sites or Rho-independent terminator signals should be nullified. However, these measurements assumed that both the mutants and the WT strains behaved in a similar manner other than the Rho-dependent termination.

We observed that terminators present upstream of the prophage genes (CP 4-6: *ykfG*, *yafZ*, *ykfI*, *yagM*, and *yagL*; Qin prophage: *ydfU*; CP 4-57: *yfjI*) are the strongest Rho-dependent terminators, which are essential to suppress the toxic prophage genes (31, 32, 33, 34). Two terminators located upstream of the tRNA genes (*thrW* and *selC)* also belonged to the very strong/strong category. They were not categorized under any group in Nadiras *et al*. 2018. An RNA folding analysis of these two genes revealed the presence of extensive secondary structures, which could have affected theirs *in vivo* characters. It has been reported that tRNA genes are highly regulated by Rho-dependent termination (29, 35). We also observed the presence of a very strong terminator in the riboswitch region of *thiC* and *thiM* (36). The functional data of these genes correlate well with our measurements of their termination efficiencies (Fig. 2*A*).

We tested how the three Rho-dependent terminators, *trpt’*, *λT*_*R1*_, and *t*_*rac*_, that are widely used in *in vitro* studies behave under *in vivo* conditions. We observed that *in vivo* efficiencies of these terminators fall under the moderate to strong categories. Interestingly, the prophage terminators are much stronger than these standard terminators.

The fold change values obtained in these RT-qPCR assays could be influenced by the *in vivo* stability and the length of the RNA transcripts used for preparing the complementary DNAs (cDNAs). We calculated the length of the RNA from the transcription start site (TSS; considering only the σ^70^ promoters that are predominantly experimentally verified as mentioned in the EcoCyc database) to the sites of the reverse primer (RP) probe of the gene (Fig. S3). Since we have harvested the mid-log phase cultures for our measurements, the majority of transcription is expected to be from σ^70^ promoters. And hence, we chose only the σ^70^ promoter-initiated transcripts in our analyses. The lengths of the theoretically calculated RNA and the free energy (ΔG) for their duplex formation did not correlate with the measured fold changes obtained in Figure 2*A* (Fig. 2, *B* and *C*). This indicated that the high RT-qPCR values did not arise from longer and more stable RNA. Next, we experimentally determined the *in vivo* stabilities of the 12 RNA transcripts transcribed from the selected genes that showed a wide range of fold change values in Figure 2*A*. We isolated RNA at different time points from the mid-log phase culture of an MG1655 strain expressing Rho N340S mutant and performed RT-PCR to quantitate the time-dependent decay of the RNA (Fig. S5, *A* and *B*) and calculated their half-lives (Fig. 2*D*). A plot of half-lives against their corresponding fold change values (obtained from Fig. 2*A*) did not show any correlation. These data strongly indicated that the fold change values described above were not influenced by the *in vivo* RNA stability and they truly represent the *in vivo* termination efficiencies of the 72 terminators. It should also be noted that the general stability of RNA transcripts in the Rho N340S strain is less than its WT counterpart (data not shown; (37)), which further supports our proposition.

### Dependence of *in vivo* termination efficiencies on the C > G bubble stretches of the rut sites

To identify and characterize the *rut* sites as well as the terminator zones upstream of the 72 genes described above, we screened for C > G bubble stretches (C-rich and G-poor sequences) using 70 nt sliding windows from 5′ to 3′ direction of the RNA (see Experimental procedures section). The C > G bubble stretches that are present in the termination zones of all the 72 genes are described in Fig. S6, *A*–*C*. Different characteristics of these bubbles could be parametrized as “descriptors” that are elaborated in Fig. S4. The values of each descriptor were obtained essentially following the methods described earlier (9) using a script written in Python (see Experimental procedures section). It should be noted that in all our analyses, the upstream most C > G bubbles are located at least 100 nt downstream of the TSSs.

Out of the 111 descriptors that were described earlier (9), we chose 13 that are directly related to the size, length, area, height, etc of the C > G bubbles and the number of YC dimers (Fig. S4 and Experimental procedures). These descriptors also best distinguish between the strong and weak terminators as obtained from the *in vitro* termination assays (9). Higher descriptor values indicate the existence of more well-defined *rut* sites. If the *in vivo* termination efficiencies are regulated by the “strength” of the *rut* sites, it is expected to observe a positive correlation between the fold change values of the 72 genes (obtained from Fig. 2) and their corresponding descriptor values.

**Figure 3:**
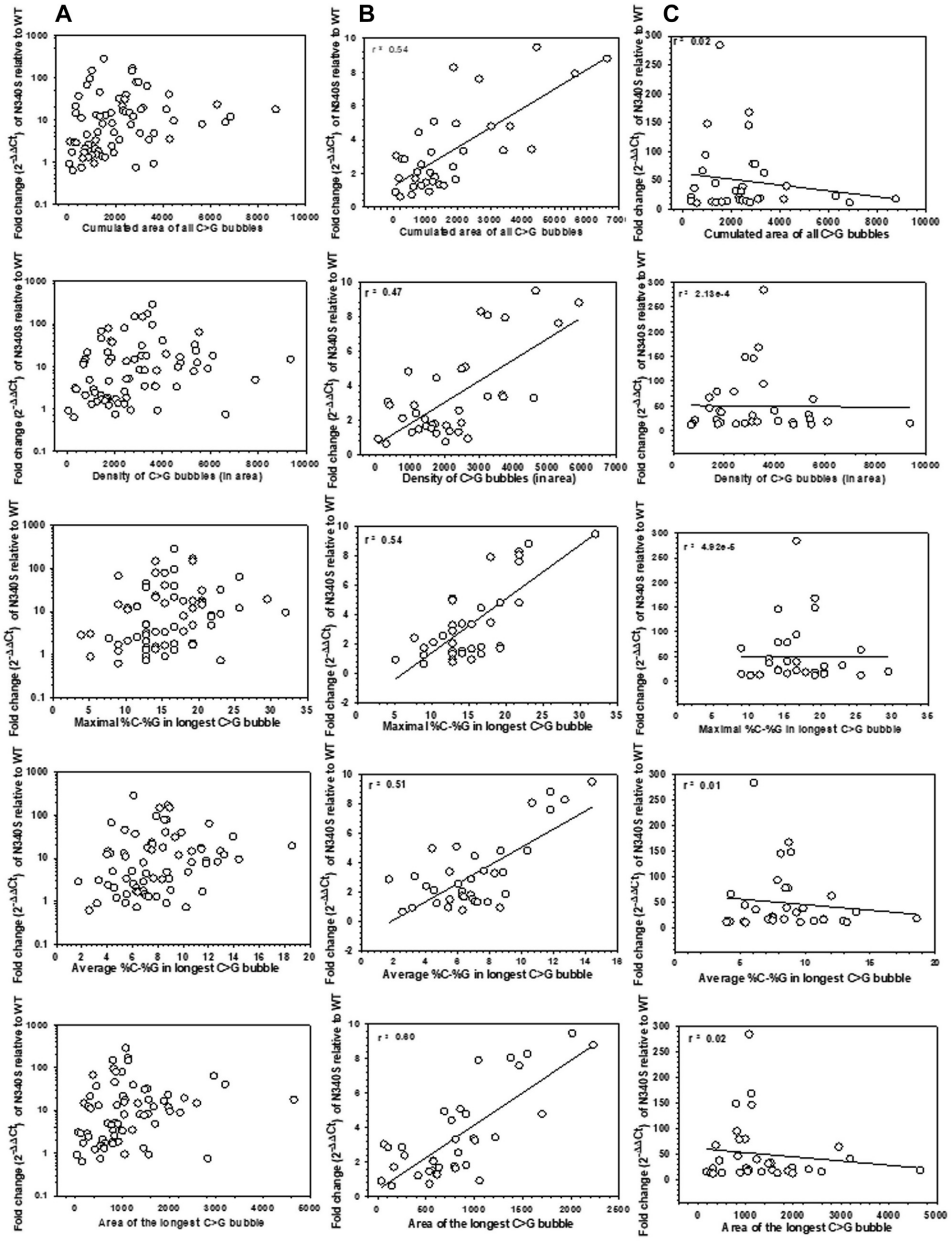
**Correlation between descriptors and the *in vivo* termination efficiencies.** The fold change values obtained from Figure 2*A* are plotted against the indicated descriptor values. In (*A*), scattered values of the fold changes (x-axis in log_10_ scale) are plotted against different descriptors, in (*B*), fold change values up to 10-fold (x-axis in linear scale) are plotted, whereas in (*C*), those with >10-fold values (x-axis in linear scale) are plotted against the indicated descriptors. In all the plots, the scattered plots were fitted to *straight lines* to establish the linear relationship and the correlation coefficients. The correlation coefficient is indicated. Calculations of values of descriptors are described in the Experimental procedures section.

A scattered plot of all the fold change values against different descriptor values appeared to lack any such correlation (Figs. 3*A*, S7*A* and S8*A*). However, when the fold change values less than 10-fold were plotted against these descriptor values, significantly high positive correlations were observed with the following descriptors: area of longest C > G bubbles (r^2^ = 0.6), cumulative area of all the C > G bubbles (r^2^ = 0.54), maximal difference (r^2^ = 0.54) and average difference between %C and %G (r^2^ = 0.51), and density of C > G bubbles (r^2^ = 0.47) (Fig. 3*B*). Other descriptors also showed moderate positive correlations (Figs. S7*B* and S8*B*). These correlations were not at all observed when > 10-fold fold change values were plotted against these descriptor values (Figs. 3*C*, S7*C*, and S8*C*). The values of descriptors did not observe to increase proportionally with the fold change values beyond a certain point. A larger C > G bubble determined by the above-mentioned descriptors offers long stretches of C-rich sequences that improve the strength of the *rut* sites for Rho-binding. And hence, these data indicated that the strength of the *rut* sites could regulate only the termination efficiencies of the weaker terminators *in vivo*. The factors other than the *rut* sequence are more important for achieving high termination efficiencies *in vivo*.

### Dependence of *in vivo* termination efficiencies on the Rho-binding strengths of the rut sites

Next, we experimentally verified whether the *rut* sites with higher C > G descriptor values have higher binding constants (K_d_) for the Rho protein (Fig. S9, *A*–*D*). We synthesized radiolabeled RNAs having the terminator zone sequences of selected genes, termination efficiencies (fold change values) of which were measured in Figure 2*A* (Fig. S9, *A* and *B*). We have synthesized >200 nt RNA sequences so that all the putative *rut* sites of a terminator zone are accommodated. These terminators have a wide range of fold change values (0.7–78 fold). We performed gel-shift assays using WT Rho to calculate the K_d_ values of each of the Rho–RNA transcript interactions (Fig. S9, *A*, *B*, *F* and *G*). The sigmoidal nature of the binding isotherms indicated the presence of multiple Rho-binding sites in these RNA transcripts (38). We used a Rho-PBS mutant, Y80C, that has reduced affinity for the *rut* sites (17), as a control. On some of the tight-binding *rut* sites, this mutant exhibited significantly reduced affinity compared to the WT (Fig. S9*G*), which indicated that the Rho-interactions with the high-affinity *rut* sites are specific. However, it should be noted that the difference between specific and nonspecific binding at the weaker *rut* sites would be much less. Upon plotting the K_d_ values against the descriptor values of the *rut* sites present in these synthesized RNA transcripts, we observed a moderate correlation (r^2^ = 0.44) with the length of the C > G bubbles (Fig. S9, *C* and *D*). This indicates that the higher descriptor value of a *rut* site could reflect higher binding affinity (lower K_d_) toward Rho. An increase in the length of the C > G bubble increases the length of C-rich sequences, and hence the binding constant increases. Interestingly, these measured K_d_ values did not show any correlation with the fold change values of the respective terminators that these *rut* sites belong to (Fig. S9*E*). This indicates that the binding strength of the *rut* sites for Rho does not influence the termination efficiency, even though these sites are required to recruit Rho to the elongation complex at some stage. Post-RNA recruitment steps such as Rho-isomerization (18) and translocation along the RNA could be more important parameters to determine the termination efficiencies. It should be noted that the *in vitro* synthesized RNA could have a different folded structure compared to the *in vivo* RNA-folding pattern, which could have contributed to the apparent mismatch between the *in vitro* binding constants and the *in vivo* termination efficiencies.

### Dependence of *in vivo* termination efficiencies on the rate of ATP hydrolysis of WT Rho

**Figure 4:**
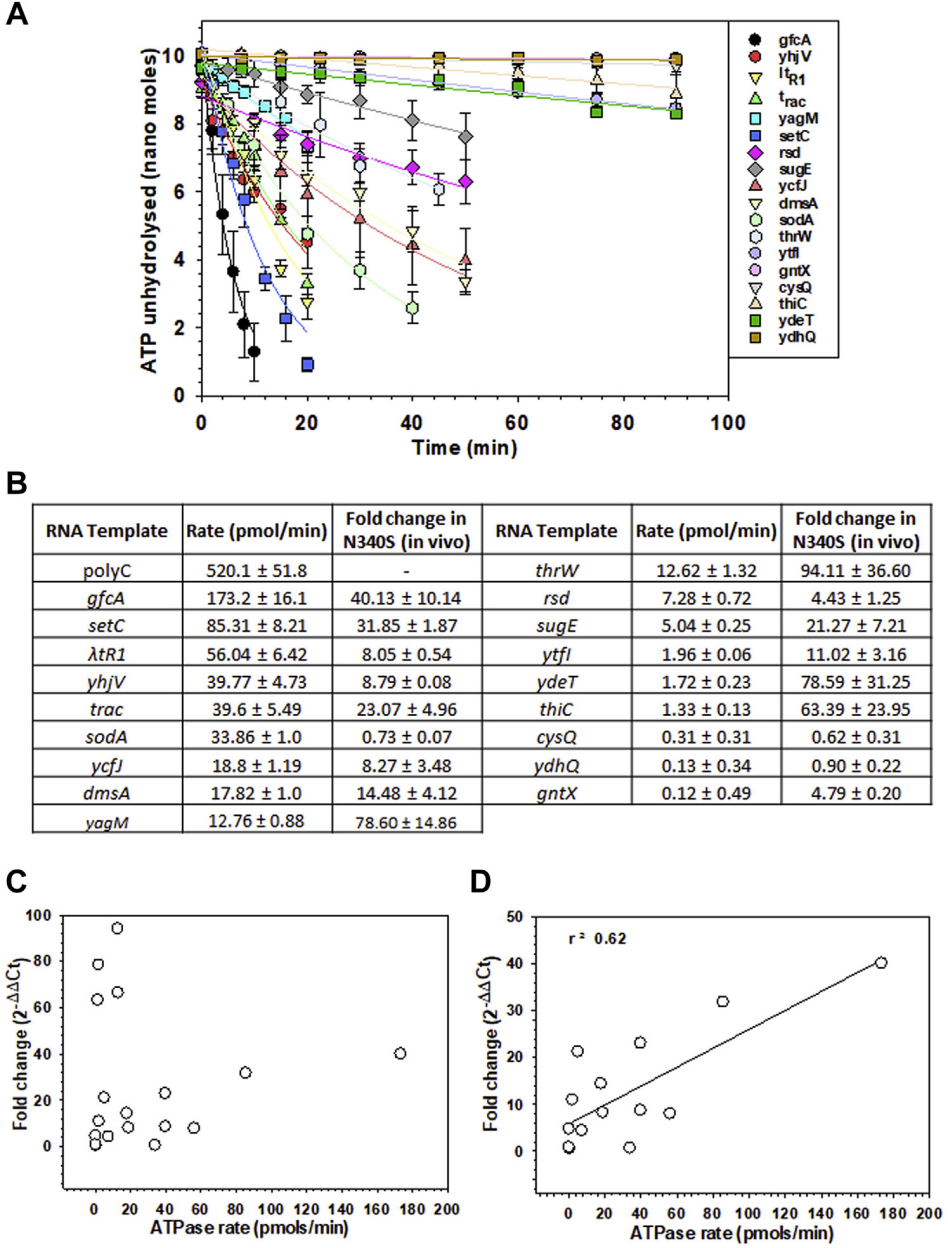
**Relationship between the rate of ATP hydrolysis and *in vivo* termination efficiencies.***A*, rates of ATP hydrolysis of Rho on different RNA templates having the terminator sequences of the indicated genes. The release of inorganic phosphate (Pi) was measured at various time points colorimetrically following the malachite green method. The rates were calculated by fitting the curves to an exponential decay equation: y = Aexp(-λt), where λ is the rate constant. *B*, a table showing the values of the rate of ATPase activity alongside their respective *in vivo* fold change values obtained from Figure 2*A*. *C*, scattered plot of fold change values against the values of rate of ATPase activity. *D*, fold change values of the terminators up to 40-fold taken from (*C*) were plotted against their corresponding rate of ATP hydrolysis. The points were fitted to a linear equation to obtain the correlation between the two parameters. The correlation coefficient value is indicated.

Rho is an efficient molecular motor that can translocate along the RNA driven by its RNA-dependent ATP hydrolysis activity, and this translocation activity has been implicated in its transcription termination function (3, 4, 6). We tested the extent of dependence of *in vivo* termination efficiency of WT Rho on its rate of translocase activities. As ATP hydrolysis is the driving force behind translocation, the rate of ATPase activity of Rho would be proportional to the rate of its translocase activity. We synthesized RNA templates having the *rut* sites of the terminator zones of several genes, *in vivo* termination efficiencies of which were measured in Figure 2, and measured the rates of ATPase activities of WT Rho on these templates (Fig. 4*A*). These templates were able to induce ATPase activity of WT Rho with a wide range of rates in the *in vitro* assays (172–0.12 pmol/min; Fig. 4*B*). The template with the sequence of terminator zones of *gfcA* yielded the highest rate that was still 3-times lower than that we achieved with the best synthetic substrate of Rho, polyC RNA. This data indicates that the Rho most likely translocates with a wide range of rates on the different nascent RNAs from different operons *in vivo*, which could have variable effects on its termination efficiencies.

We next plotted these rates of ATPase activities against the fold change values of the corresponding genes (see Fig. 2*A*) from which these RNA transcripts were prepared. We observed a positive correlation (r^2^ = 0.62) between these two parameters for most of the terminators, except those having very high *in vivo* termination efficiencies (fold change values >40-fold; *yagM, sugE, ydeT*, and *thiC*; Fig. 4, *C* and *D*). Among them, the *thiC* terminator is part of the thymine pyrophosphate–activated riboswitch (36), which might have affected its termination efficiency in addition to the translocase rate. These data strongly indicate that in general, the *in vivo* termination efficiency is dependent on the rate of translocase activity of Rho, and the RNA sequences of the terminator zone induce variable translocation speed. However, terminators with very high termination efficiency could also be under the control of *in vivo* factors in addition to the rate of translocase activity.

### Dependence of rate of ATPase activities on the descriptor values

**Figure 5:**
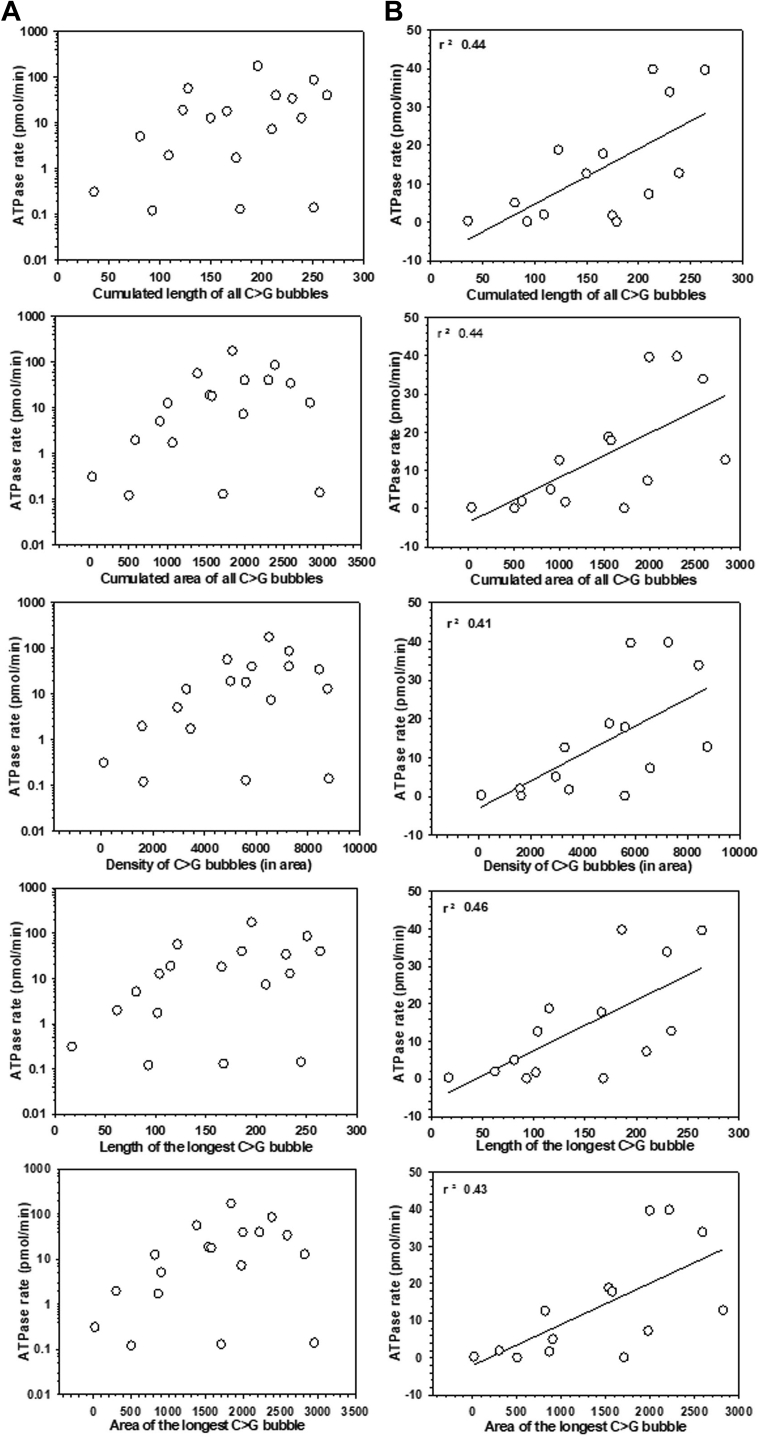
**Relationship between rates of ATPase activity and the descriptor values.** In (*A*), scattered plots of rates of ATPase (x-axis in log_10_ scale) activities against the indicated descriptor values, and in (*B*), the rates of ATPase activities (x-axis in linear scale) up to 50 pmol/min were plotted against the indicated descriptor values and were fitted to a linear equation to obtain the correlation coefficients. The correlation coefficients are indicated in each plot.

Next, we explored the dependence of the rate of ATPase activity on the C > G bubble sequence patterns of the *rut* sites. This also enabled us to understand how the descriptor values of the *rut* sequences influence the Rho-termination efficiency *via* controlling the translocase activity. Upon plotting the rates of ATPase activities (shown in Fig. 4) against the descriptor values of the corresponding terminators, we observed a moderately good positive correlation (r^2^ > 0.4) with the descriptor values that describe the area and length of the C > G bubbles (Fig. 5, *A* and *B*). This correlation was observed for the ATPase rates < 50 pmol/min. The highest correlation was observed with the descriptor that defines the length of the longest C > G bubble. The descriptors defining other parameters of the C > G bubbles did not show good correlations (Figs. S10 and S11). These data indicate that the C > G bubble patterns of the rut sites can influence the ATPase rates to a certain degree (up to 50 pmole/min). As the rate of ATP hydrolysis is dependent on the Rho-binding and isomerization on the *rut* sites, this correlation is expected. These two steps are rate-limiting and are dependent on the C > G bubble sequences. Hence, the bubble sequences primarily influence the rate of ATPase *via* these initial steps. However, a very high rate of ATPase activity on certain terminators like *gfcA*, *setC*, etc, could be dependent on the postrecruitment steps such as the presence of duplex structures or the presence of long unhindered stretches of single-stranded regions on the RNA transcripts encountered by Rho during the translocation.

### Effects of intrinsic pause sequences on the Rho-dependent termination efficiencies

So far, we have described the dependence of the *in vivo* termination efficiencies on the Rho–*rut* interaction, Rho translocation, and the sequence patterns of the C > G bubbles. However, the very high termination efficiencies of certain terminators did not correlate with these parameters, indicating their regulation by other factors. The transcription EC faces numerous pause sites that slow down its rate of elongation, which could facilitate the Rho-dependent termination efficiency. We reasoned that the EC could encounter pause sequences in the genes that allowed Rho to function with very high termination efficiencies (>30 fold, Fig. 2). Using the database that describes the elemental pause sequences, GGcataatTG(C/T)GGCcg (39), we found that these pause sites are present in *thiC*, *sbp*, and *yegQ* (Fig. S12). We inferred that these pause sequences might have contributed to their respective termination efficiencies in addition to the above-mentioned nascent RNA-dependent parameters.

### NusG dependency of the *in vivo* termination efficiencies of various Rho-dependent terminators

**Figure 6:**
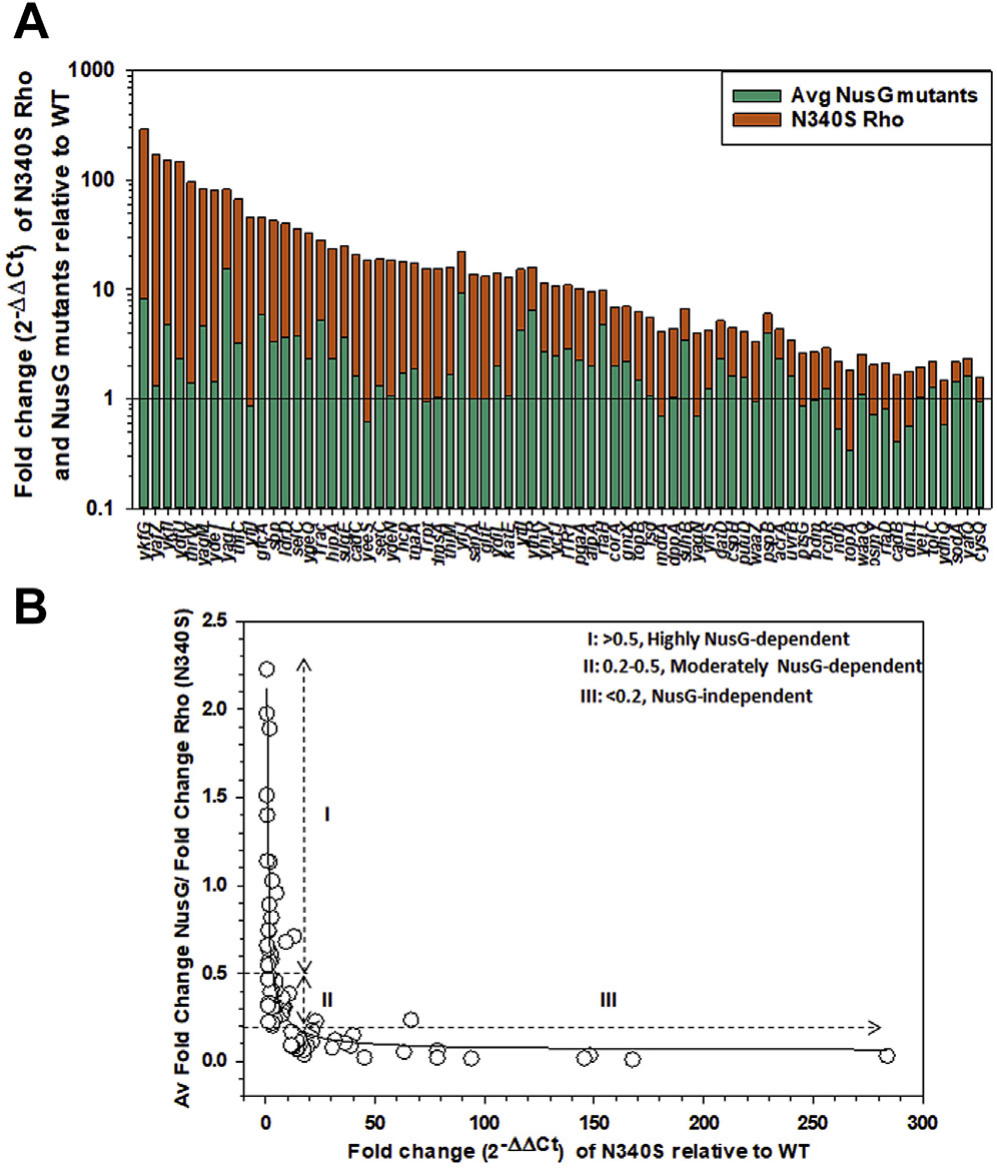
**NusG-dependent terminators a subset of Rho-dependent terminators.***A*, a stacked plot showing the relative RT-qPCR values (fold-change relative to WT) obtained for the indicated genes in the Rho N340S mutants stacked upon the corresponding average fold change (relative to WT) obtained from the three NusG mutants (G146D, V160N, L158Q). Fold change values for each of the NusG mutants are shown in Fig. S13. *B*, the ratios of average fold change values obtained from the NusG mutants (numerator) and that obtained from the N340S Rho (denominator) are plotted against the relative fold change values (relative to WT) obtained from the Rho N340S mutant to identify the NusG-dependent terminators. Three categories of terminators based on their NusG-dependence are indicated.

A subset of Rho-dependent terminators is dependent on NusG (10). We explored the *in vivo* NusG-dependence of the Rho-dependent terminators associated with 72 genes that we described in Figure 2*A* and the existence of NusG-specific signature (s) in the C > G bubble sequences of these terminators. We chose MG1655 strains that express the Rho-binding defective NusG mutants, G146D, V160N, and L158Q (14) that cause reduced *in vivo* termination efficiency of Rho. We measured the gene expressions of these genes in the NusG mutants using RT-qPCR and calculated the fold changes in gene expression relative to the WT strain in the same way as described in Figure 2 (Fig. S13, *A* and *B*). We calculated the average fold change values from the fold change data of all the three NusG-mutants (Fig. 6*A*).

A wide range of average fold change values of the 72 genes was obtained in the presence of the NusG mutants (Figs. 6*A*, S13, *A* and *B*). However, the values were less compared to that obtained in the presence of the Rho mutants, N340S (compare with Fig. 2*A*), and also the NusG- and Rho-mutant data sets did not follow a linear correlation. In general, for the strong terminators, the fold change values obtained in the Rho mutant are much higher than that observed in the NusG mutants (*e.g.*, *yagL* showed 15.6 fold in the NusG mutants and 66.6 fold in the Rho mutant). But in cases of weak or moderately strong terminators, these differences in the upregulation (fold change) were observed to be much less. As NusG-dependent terminators are a subset of Rho-dependent terminators and the NusG dependency of all the Rho-dependent terminators varies considerably (Figs. 6*A* and S13), we reasoned that a quantitative measure of the *in vivo* NusG-dependency would be obtained properly if the ratios of average fold change obtained in the NusG mutants to that in N340S Rho are plotted against the fold changes obtained in the N340S Rho mutants (Fig. 6*B*). The plot showed a biphasic nature, where the ratio of fold-change values > 0.5 was considered to be highly NusG-dependent (21 genes). Values within 0.2 to 0.5 were classified as moderately NusG-dependent (31 genes) and the values < 0.2 were classified as NusG-independent (20 genes). Most of the terminators that are categorized as none or weak terminators in Figure 2*A* were also observed to be highly NusG dependent *in vivo*. Hence, the nature of *in vivo* NusG dependency of these 72 terminators was consistent with the mechanism of NusG action proposed from the *in vitro* studies (18, 19).

### Signatures of NusG-dependent terminators

**Figure 7:**
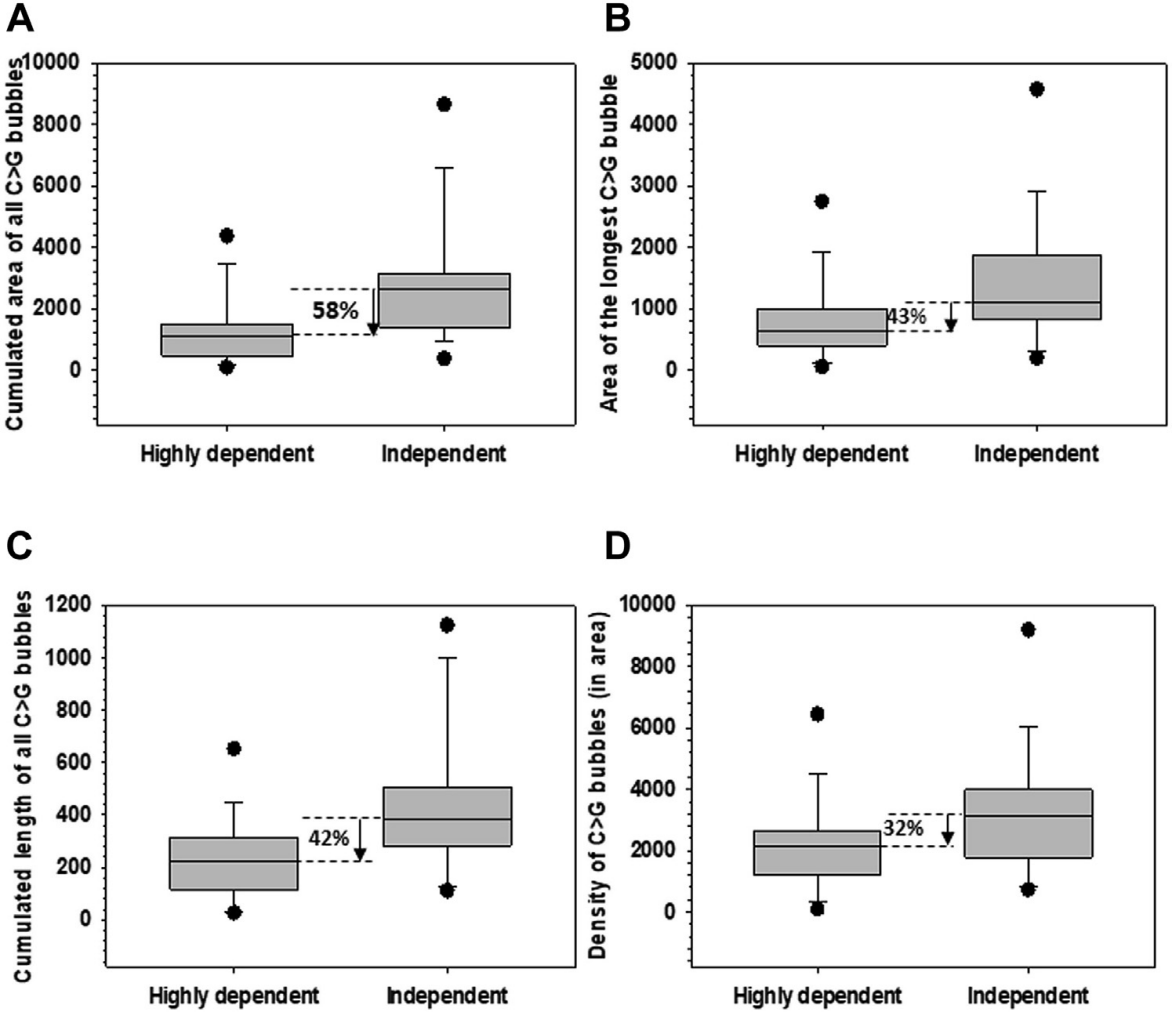
**Signatures of NusG dependent terminators.***A*–*D*, boxed plots showing the mean differences in indicated descriptor values between highly NusG dependent and independent groups (Fig. 6*B*). *A*, cumulated area of all C > G bubbles shows a reduction of 58%, (*B*) area of the longest C > G bubble shows a reduction of 43%, (*C*) cumulated length of all C > G bubbles shows a reduction of 42%, and (*D*) density of C > G bubbles (in the area) shows a reduction of 32% in the highly NusG-dependent group. The highly NusG-dependent group includes the terminators that have ratios of average fold change values obtained from the NusG mutants and that obtained from the N340S Rho > 0.5 (obtained from Fig. 6*B*).

It is not clear which *rut* site characteristics are important for a Rho-dependent terminator to become NusG-dependent. So, we explored the descriptor values of different C > G bubble stretches of the NusG-dependent terminators obtained from Figure 6*B* to identify their *rut*-site signatures. We analyzed the distribution of different descriptor values for all the NusG-dependent and NusG-independent terminators and expressed them in the form of box plots (Figs. 7, *A*–*D* and S14). We observed a significant difference in the mean and median of some of the descriptor values between the NusG-dependent and NusG-independent categories. The most prominent difference was observed in four descriptors: cumulated area of all C > G bubbles, area of the longest C > G bubble, cumulated length of all C > G bubbles, and density of C > G bubbles (in the area) (Fig. 7). We expressed the difference in terms of percentage reduction in the median between the two categories. In all the cases, the median values reduced significantly in the NusG-dependent terminators. The maximum difference between the two categories was seen in the cumulated area of all C > G bubbles (58%). We concluded that the NusG dependent terminators, in general, have *rut* sites with the reduced area, length, and density of the C > G bubbles. A Rho-dependent terminator with lower termination efficiencies and marked by *rut* sites with diminished C > G bubble patterns should be a likely candidate to be a NusG-dependent terminator. Our conclusion is consistent with what was proposed earlier (10).

## Discussion

Even though detailed analyses of the *in vitro* mechanism of bacterial Rho-dependent transcription termination have been reported (1, 3, 4, 6), understanding of this termination process *in vivo* is limited. Here, we measured the *in vivo* termination efficiencies of 72 Rho-dependent terminators in the mid-log phase cultures of *E. coli* strains that express either a Rho mutant (N340S) or NusG mutants (G146D, L158Q, and V160N) and established their correlation or lack of it with the C > G bubble patterns of their respective *rut* sites, binding constants of Rho–*rut* site interactions, the Rho translocase activity on the nascent RNA, and *in vivo* NusG-dependence of these terminators. We made the following observations. (1) These terminators exhibited a wide range of terminator strengths *in vivo* where the prophage terminators exhibited to be the strongest ones. Many of the terminators behaved differently *in vivo* compared to that predicted or experimentally shown *in vitro* (Fig. 2). (2) Only the weaker terminators under *in vivo* conditions showed a positive and robust correlation with the nature (size, length, density, etc) of the C > G bubble stretches of the *rut* sites. This correlation disappeared as the terminator strength increased suggesting a limited role of the nature of *rut* sequences in controlling the termination efficiencies *in vivo* (Figs. 3, S7, and S8). This observation was despite the fact that the intrinsic binding affinities of Rho for the *rut* sites were dependent on the nature of the C > G bubbles (Fig. S9). (3) The *in vivo* termination efficiencies of the majority of the terminators showed good correlations with the rates of ATPase activities of Rho on the RNA templates made of the sequence of the respective terminator-zones (Figs. 4, S10 and S11) indicating the Rho-translocase activity-dependence of the terminators *in vivo*. (4) Consistent with the earlier propositions (18, 19), the weaker Rho-dependent terminators *in vivo* showed significant dependence on NusG (Figs. 6 and S13). (5) The signatures of highly NusG-dependent terminators are marked by reduced area, length, and density of the C > G bubbles, the pattern which could be used to predict unknown NusG-dependent terminators from the genome sequence (Figs. 7 and S14). We concluded that Rho-dependent termination follows a nascent-RNA dependent pathway *in vivo*, where Rho translocation along the RNA is essential and *rut* sequences may be required to recruit Rho but the binding strengths of the latter for the *rut* sites have limited influence on the outcome of the termination events.

Based on the *in vitro* Rho-dependent transcription termination assays, two types of termination pathways have been proposed: RNA-dependent and RNAP-dependent pathways (1). In the RNA-dependent pathway, Rho loads onto the *rut* sites of the nascent RNA and following translocation along with the RNA catches up the EC before dislodging the latter *via* a direct interaction. In the more recently reported RNAP-dependent pathway, Rho is directly recruited onto the RNAP of the EC, inactivates the EC, and terminates the transcription (25, 27). In this model, Rho may be transferred to the exiting RNA after loading onto the EC. In the recently solved EC–Rho complex structures, Rho-induced catalytically inactivated state(s) of RNAP was observed, which was coined as a preterminated state(s)of the EC (26, 28). As these inactivated ECs were observed in the absence of ATP hydrolysis function of Rho, the requirement of Rho-translocation for its termination function was questioned. Here, we show that the *in vivo* termination efficiencies of the majority of the 72 terminators are correlated and dependent on the Rho ATPase as well as its translocase function. However, the *in vivo* termination efficiencies, especially at the genes where the efficiency values are high, are not dependent on the binding strength of the *rut* sites for Rho. Our results support the model that Rho follows the RNA-dependent pathway to execute the *in vivo* transcription termination. To this end, we cannot rule out the possibility that the EC might play an anchor to gravitate the Rho protein onto the nascent RNA and thereby facilitate the Rho-recruitment process *in vivo*.

*In vitro* analyses suggested that NusG-CTD-Rho facilitates the conversion of open to close complex formation of Rho at the *rut* sites of a subset of terminators (18, 19). However, in the recently solved Rho-EC structures (26, 28), the NusG-CTD was not visible indicating its lack of stable interaction with Rho when they are part of an EC. Our *in vivo* termination data show that NusG-dependence is highly pronounced for the terminators that have weak termination efficiencies and the size, length, area, etc. of the C > G bubble of their *rut* sites are significantly diminished. A reduced C > G bubble stretch could pose a higher activation energy barrier for the isomerization steps at the *rut* sites, and NusG reduces this energy barrier. Hence, our *in vivo* functional data is inconsistent with the Rho-EC structure, rather it supports the proposed mechanism of NusG action in the Rho-dependent termination (18, 19) and further supports the existence of an RNA-dependent pathway of termination *in vivo*.

## Experimental procedures

### Materials

[γ-^32^P] ATP (3000 Ci/mmol) and [α-^32^P] UTP (3000 Ci/mmol) were from Jonaki, BRIT. Antibiotics, lysozyme, DTT, and bovine serum albumin were from USB. Primers for PCR and dC_34_ were obtained from Eurofins. Restriction endonucleases, T4 polynucleotide kinase, and T4 DNA ligase were from New England Biolabs. Taq DNA polymerase was from Roche Applied Science. RNAlater used for storing RNA samples used in microarray experiments were from Ambion. RNAeasy kit and RNAprotect Bacteria used for RNA isolation were from Qiagen. DNase I amplification grade, Superscript III-RT, RNase out, EDTA, DTT, and random hexamers were purchased from Invitrogen. Twenty millimolar MgCl_2_ and ten millimolar dNTP solutions were from Thermo Scientific. dATP and poly(C) RNA were obtained from Amersham. TB Green Premix Ex Taq II (Tli RNase H Plus) from Takara was used for RT-qPCR. Sodium-deoxycholate and Phenol were from USB. AmpliScribe T7 High Yield Transcription Kit was purchased from Lucigen. Malachite green (MG), ammonium molybdate, and polyvinyl alcohol were obtained from Sigma. All the bacterial growth media were from Difco.

### Bacterial strains

RS862 used to study the properties of the Rho and NusG mutants is a derivative of *E. coli* MG1655, in which chromosomal *rac* prophage was deleted by P1 transduction (*rac::tet*^*R*^). The *rac* prophage was deleted since it contains the *kil* gene, expression of which causes lethality in the termination-defective Rho mutants. This resultant strain was transformed with pCL1920 expressing either WT or the mutant derivatives of Rho (N340S) or NusG (G146D, V160N, and L158Q) proteins (see Fig. S1). Finally, the chromosomal *rho* and *nusG* were deleted by P1 transduction using the Keio collection (*rho::kan*^*R*^; *nusG:: kan*^*R*^).

To measure the *in vivo* termination efficiencies of the standard terminators, λ*T*_*R1,*_ *trpt’*, and *t*_*rac*_, RS1879 was transduced with the constructs *P*_*lac*_*-trpt’*-*lac* ZYA, *P*_*RM*_*-t*_*rac*_*-lac* ZYA, and *P*_*lac*_*-λT*_*R1*_*-lac* ZYA using the phage λRS45.

Description of all the strains and plasmids used in this study are in Table 1. All the primers used in the study are described in Table S1.

**Table 1:** List of strains and plasmids used

Strains	Description	Reference
DH5a	*Δ(argF-lac)U169 supE44 hsdR17 recA1 endA1 gyrA96thi-1 relA1 (ø80lacZ*ΔM15)	
MG1655	WT	
RS330	GJ3161*Δrho:: Kan*^*R*^, with pHYD1201 shelter plasmid, *Amp*^*R*^ RS257	(43)
RS331	GJ3161*ΔnusG:: Kan*^*R*^, with pHYD1201 shelter plasmid, *Amp*^*R*^ RS257	(43)
RS862	MG1655 WT *Δrac* strain	(30)
RS1305	MG1655*Δrac* carrying marker less Δ*rho* (*Δrho::FRT*) with pHYD1201 *Amp*^*R*^ (AI)	(18)
RS2045	MG1655, *Δrac, Δlac*, λRS45 lysogen carrying P*lac-trpt’ -lacZYA*	(44)
RS2046	MG1655, *Δrac, Δlac*, λRS45 lysogen carrying P*lac-t*_*rac*_*-lacZYA*	(44)
RS2047	MG1655, *Δrac, Δlac*, λRS45 lysogen carrying P*lac-t*_*R1*_*-lacZYA*	(44)
Plasmids		
pHYD1201	3.3 kb HindIII-SalI fragment carrying rho+subcloned from pHYD567 into HindIII/SalI sites of pAM34 (pMB1; IPTG-dependent replicon, *Amp*^*R*^)	(43)
RS96	pET21b with WT *rho* cloned at NdeI/XhoI site, His-tag at C-terminal; *Amp*^*R*^	(17)
pRS317	pHYD567-3.3 kb NsiI fragment carrying *rho+* cloned from λ phage 556 of Kohara library into PstI site of pCL1920 (pSC101; *Sp*^*R*^*,Sm*^*R*^	(43)
pRS316	pHYD547-3.8 kb chromosomal SmaI fragment carrying nusG cloned into SmaI site of pCL1920 (pSC101; *Sp*^*R*^*,Sm*^*R*^)	(43)
pRS344	pHYD567-*rho* G324D, *Sp*^*R*^*,Sm*^*R*^	(17)
pRS346	pHYD567-*rho* N340S, *Sp*^*R*^*,Sm*^*R*^	(17)
pRS944	pHYD547-*nusG* L158Q, *Sp*^*R*^*,Sm*^*R*^	(21)
pRS1047	pHYD547-*nusG* V160 N, *Sp*^*R*^*,Sm*^*R*^	(21)
pRS1049	pHYD547-*nusG* G146D, *Sp*^*R*^*,Sm*^*R*^	(21)
pRS1865	pHYD547-*nusG* L158Q, *Sp*^*R*^*,Sm*^*R*^	(21)

### Microarray analyses

The RS862 was at first transformed with pCL1920 plasmids expressing either WT or the mutant derivatives of Rho and NusG, and subsequently, the chromosomal WT copies of Rho and NusG were deleted by P1 transduction. Overnight cultures of these strains were subcultured in 10 ml LB with appropriate antibiotics and were allowed to grow until A_600_ ∼ 0.3 to 0.4. The culture was spun down and the cell pellet was resuspended in 1 ml of RNAlater. RNA isolation and the microarray experiments were performed by Genotypic Technology as described in an earlier publication from our laboratory (30). Fold changes in gene expression for each strain were calculated relative to WT Rho or WT NusG, respectively.

### RNA purification and qPCR reaction

The RS862 was transformed with pCL1920 expressing WT and the mutant derivatives of Rho and NusG followed by deletion of chromosomal copies of these two genes. RNA was isolated from mid-log phase (A_600_ ∼ 0.3–0.4) cultures of these strains using Qiagen’s RNAeasy kit, following which the residual genomic DNA in the RNA preparations was removed by the DNase I treatment. One microgram RNA was used for the synthesis of cDNA using Superscript III Reverse Transcriptase following the standard procedures. The amount of cDNA produced during the PCR cycles was monitored in real-time using SYBR green dye in the Bio-Rad CFX96 RT-PCR system. The threshold cycle ‘Ct’ was calculated from the midpoint of the sigmoidal curve obtained by plotting the fluorescence intensity against the number of PCR cycles. 2^–ΔΔCt^ was used to calculate the fold change in the mRNA level for the mutants relative to the WT, where Ct = the number of threshold cycles; ΔCt = [Ct of target gene] – [Ct of internal control]; and ΔΔCt = [ΔCt of mutant] – [ΔCt of WT]. The level of *rpoC* mRNA was used as an internal control. Primer pairs were designed to probe various regions of an operon so that the PCR products were within 200 nt in size. For the terminators, *t*_*rac*_, *trpt’*, and *λt*_*R1*_, the primer probes were in lacZ gene fused downstream to them.

### Sequence selection and analyses of rut sites

Reference genomes used for bioinformatics analysis is the *E. coli* genome U00096.3. The selection of sequences was done based on qPCR analysis. Multiple primer probes were designed for each operon that was upregulated in our microarray data profiles (GEO ID: 200077176 and 200077177; see Fig. S2; list of primers is given in Table S1) and those described in a study using global RNA profiling techniques (10) in the presence of Rho and NusG mutants and upon treatment with bicyclomycin. Few operons that are under the control of Rho-dependent termination were also chosen from the *in vitro* study of Rho-dependent terminators ((9); see Fig. S2). The sequences in the genes for primer binding were selected based on the following: if the upregulation was observed in the first gene of an operon, the sequences from the TSS (from the EcoCyc database, we considered the experimentally determined promoters) till the end of the RP probes inside the first gene were taken for analysis, whereas if upregulation was seen in the genes downstream of the first gene, the sequences from the RP probe of the downstream gene to the RP of the upstream gene was taken for analysis (see Fig. S3). Sequences were checked for the presence of %C > %G bubbles using a python script and calculating the %C and %G using a sliding window of 70 nt with 10 nt increment along with the selected sequences. RNA folding free energies were determined with the Vienna RNA folding software (40) using default settings with a window size of 70 nt.

The descriptor values were calculated using the python script “MakeDescriptors” as described in Nadiras *et al.*, 2018. The script analyses certain sequence characteristics named “descriptors” and gives a value. It uses a sliding window of 78 nt on the sequence for the calculation of descriptor values such as %C and %G (Figs. S4 and S6). Among the various descriptors (111 descriptors) described in Nadiras *et al*, 2018, we chose descriptors that are related to C > G bubbles and were among the ‘most differentiating’ ones. These chosen descriptors are length of the longest C > G bubble, cumulated length of all C > G bubbles, density of C > G bubbles (along the sequence), area of the longest C > G bubble, cumulated area of all C > G bubbles, area density of C > G bubbles, maximum %C in a longest C > G bubble, maximal %C - %G in a longest C > G bubble, average %C - %G in a longest C > G bubble, number of [(YC)N9→13]1 motif (YC dimers) in longest C > G bubble, number of [(YC)N9→13]2 motifs in longest C > G bubble, number of [(YC)N9→13] 3 motifs in longest C > G bubble, and the density of [(YC)N9→13]1 motif (%YC) (see Fig. S6 for the details). The meaning of each descriptor is as follows: The length of the longest C > G bubble calculates the length of the longest bubble (L1). The cumulated length of all C > G bubbles calculates the sum of all C > G bubbles' length (L1+L2) within the sequence of 78 nt window. The density of C > G bubbles (along the length) describes the fraction of cumulated length of all C > G bubbles relative to the total length of the sequence (Lt) ((L1+L2)/Lt). The area of a bubble (Si in Fig. S6) was determined by summing up %C - %G values over the length of the bubble (area are thus expressed in % x bp). The area of the longest C > G bubble describes the area that comes under the longest C > G bubble (S1). The cumulated area of all C > G bubbles describes the sum of the area under all C > G bubbles (S1+S2). The area density of C > G bubbles describes the fraction of the cumulated area of all C > G bubbles along the length of the sequence under study (S1+S2)/Lt). Maximal %C in the longest C > G bubble describes the maximum %C value among all the 78 nt sequence windows within the longest C > G bubble. Maximum %C - %G in the longest C > G bubble describes the point of largest difference between the %C and %G ((C-G)max% Bub). Average %C - %G in the longest C > G bubble describes the average of the difference between %C and %G at each point within a C > G bubble (C-G) av % Bub). Structural data suggested that 5′-YC (Y = pyrimidine) dimer sequences in the G-C stretches are important for Rho-PBS-RNA binding. The number of [(YC)N9→13] 1/2/3 motifs in the longest C > G bubble counts the numbers of YC dimer motifs present inside the longest C > G bubble. When the dimer motifs are repeated, the distances between the two consecutive motifs will be 9 to 13 bases. Number of [(YC)N9→13] 1 counts all YC dimers. [(YC)N9→13] 2 counts the occurrence of two consecutive YC dimers. [(YC)N9→13] 3 counts the occurrence of three consecutive YC dimers. The density of [(YC)N9→13]1 motif (%YC) calculates the fraction of the number of YC dimers within the length of the sequence under study ((Number of [(YC)N9→13]1)/L_t_).

### Calculation of correlation coefficients

In all the correlation graphs, scattered points were plotted using SigmaPlot 13 and they were fitted to straight lines by linear regression analyses using the formula (y = y_0_ + ax). The goodness of fit for the linear regression (r^2^) values was also calculated using Sigmaplot 13 and the probability of a hypothesis (*p*-value) was calculated using GraphPad Prism. The plots with higher correlation coefficients (r^2^ > 0.3) had *p*-values in the range of > 0.0001 to 0.041. Linear regression plots with lower r^2^ values were found to have higher *p*-values (>0.05) and hence considered to be not significant.

### Box plot analyses of the distribution of descriptors

Box plots were used to represent the difference in the range of descriptor values of different terminator classes. The Box plot was plotted in Sigmaplot 13 using linear interpolation to determine the percentile value and computing the median of the data that is represented by a straight line in the box. Data was divided into four quartiles, each having 25% of the data. Outliers (10th percentile and 25th percentile) are represented as dotted symbols.

### Templates for *in vitro* binding assay and *in vitro* ATPase assay

Different linear DNA templates for RNA synthesis containing the predicted *rut* site sequences were synthesized by PCR in such a way that in all the cases, transcription starts from T7 φ10 promoter. This promoter sequence was incorporated in the forward primers. The reverse primer was designed in such a way that all the PCR products were ∼300 bp in length. The PCR amplifications were performed using high fidelity Deep Vent polymerase from NEB and the chromosomal DNA was used as a template for the PCR reactions. These templates were transcribed using the AmpliScribe T7 High Yield Transcription Kit following the manufacturer’s protocol. For the Rho-RNA binding assays, the RNAs were labeled with [α-^32^P] UTP during the synthesis. For the ATPase assays, the RNAs were synthesized in the same way, except that radioactive NTP was not used. The poly (dC_34_) was labeled with [γ-^32^P] ATP using T4 polynucleotide kinase.

### Binding assay

WT Rho-RNA binding constants were measured by gel-shift assays using radio-labeled RNA. In each assays, 5 nM of RNA was used in the transcription buffer (25 mM Tris–HCl (pH 8.0), 5 mM MgCl_2_, 50 mM KCl, 1 mM DTT and 0.1 mg/ml of bovine serum albumin) with the increasing concentrations of the WT Rho (0–50 nM). The binding reactions were performed at 37 °C for 5 min before loading onto a running 4% (w/v) native acrylamide gel under cold (4 °C). Gels were scanned in phosphor-imager Typhoon 9200 and fractions of unbound species were quantified by ImageQuant software. Gel-shift assays with radio-labeled polydC_34_ were also performed in the same way. The dissociation constant (K_d_) values were calculated by fitting the binding curves to either sigmoidal or hyperbolic equations using the SigmaPlot 13.

### ATPase assay

The colorimetric determination of ATPase activity was performed by an MG assay using the procedure previously described in an *in vitro* ATPase study (41) with slight modification. The MG reagent was freshly prepared every day and consisted of a mixture of MG solution (0.00812%, w/v in water), polyvinyl alcohol aqueous solution (2.32%, w/v), ammonium molybdate (5.72%, w/v) in 6M HCl, and water in 2:1:1:2 ratios. The MG reagent was left standing for 3 h to get a stable green/golden solution, which was filtered through a 0.45 μm filter before use. All the ATPase assays were performed in the transcription buffer. The release of inorganic phosphate was measured at various time points colorimetrically by the MG method. ATPase reaction was stopped by the addition of 50 μl MG solution at each time point, and volume was made up to 196.875 μl by adding 136.875 μl of assay buffer and incubated for 1 min and then 3.125 μl of sodium citrate aqueous solution (34% w/v) to limit ATP hydrolysis (42). The solution mixture was incubated for 30 min, following which absorbance was measured at A_630_.

### *In vivo* RNA stability assays

*E. coli* WT strain, MG1655Δ*racΔrho* having pCL1920 plasmid expressing N340S Rho was grown in LB broth at 37 °C until the early-log phase (A_600_ ∼ 0.3–0.4). Two milliliters of culture was harvested (time point 0) and immediately RNAprotect Bacteria (Qiagen) were added to stabilize the RNA *in vivo*. At that point, transcription was stopped by treatment with 50 μg/ml rifampicin and the cells were incubated for an additional 2, 4, 6, 8, and 10 min, respectively, following which cells were harvested and were resuspended in RNAprotect Bacteria, and RNA was isolated using the Qiagen RNeasy Kits. Residual genomic DNA in the RNA preparations was eliminated by DNase I treatment. One microgram RNA was used for the synthesis of cDNA using Superscript III Reverse Transcriptase following the standard procedures. The cDNA was used for RT-PCR amplification for estimating the amount of RNA left at different time points. PCR products were run on 1.5% agarose gels that were visualized on Alpha Imager Gel Imaging System. The decrease of DNA band intensities relative to the control (time point 0) was estimated by ImageQuant software. Band intensities were plotted and the curves were fitted to an exponential decay equation to calculate the half-life and degradation constants of the RNAs *in vivo*.

## Data availability

All the raw datasets are available upon request. Computer codes (in python script) are written based on that described in Nadiras *et al*., 2018 and are available upon request.

## Supporting information

This article contains supporting information (9, 10, 17, 30, 39).

## Conflicts of interest

The authors do not declare any competing interests. P. I. R. C. is a DBT Senior Research fellow.
