# Supplementary material for: In vivo regulation of bacterial Rho-dependent transcription termination by the nascent RNA
Source: J Biol Chem. 2022 Apr 29;298(6):102001. doi: 10.1016/j.jbc.2022.102001 (PMC9160355; doi:10.1016/j.jbc.2022.102001)
Supplement: Supplemental Figures S1–S14 and Table S1 [file mmc1.pdf]

Fold change of the gene expression values in the presence of the Rho or NusG mutants or Bicyclomicin (BCM) relative to that in the presence of WT or in the absence of BCM obtained from Microarray profiles (Shashni et al, 2014) or from the RNA-seq analyses (Peters et al., 2012)

Gene Name	*nusG* G146D	*nusG* V160N	*nusG* L158Q	*rho* N340S	BCM
cadA	2.06	3.01	2.42	4.96	2.58
cadB	1.66	2.38	2.07	2.5	1.63
cadC	0.94	0.94	1.86	0.63	1.88
dppA	-0.52	-0.18	2.67	-0.14	-0.53
dppB	0.21	0.76	4.03	0.67	1.02
gfcA	2.18	1.633	2.164	1.62	2.224
gfcB	3.37	2.61	2.84	3.2	4.17
gfcC	3.61	2.6	0.66	5.75	3.1
gltF	1.28	1.48	1.88	2.42	2.79
hipA	0.33	0.31	0.93	0.04	-0.27
rem	0.54	0.41	0.08	-0.52	0.04
rfaH	0.75	0.68	0.86	0.24	-0.81
sanA	1.36	0.72	3.77	0.29	1.19
sbp	0.69	0.76	3.54	0.41	0.45
suhB	1.6	1.48	0.9	0.51	-0.51
yfhR	1.974	1.866	1.932	3.899	4.545
rcnR	0.021	0.109	-0.51	-0.87	0.182
thiM	2	2.02	0.86	1.56	1.83
thiC	1.83	2.49	2.47	5.68	3.43
setC	2.02	2.55	1.32	2.01	3.26
thrW	0.11	0.06	0.53	-0.32	0.04
yddA	2.47	2.42	-0.42	0.69	2.59
yagM	0.727	0.431	0.938	4.187	4.301
yagL	3.19	2.93	2	5.65	4.65
sugE	1.09	0.94	1.25	1.59	0.39
ydeS	2.24	2.18	0.48	3.63	4.45
tolC	-0.22	-0.03	-0.44	-0.93	-1.27
acrA	0.39	0.39	-0.25	-0.06	-0.82
acrB	0.06	0.3	-0.79	-0.55	-1.18

Gene Name	*nusG* G146D	*nusG* V160N	*nusG* L158Q	*rho* N340S	BCM
yafZ	1.29	0.86	0.08	0.4	0.58
yafY	0.403	-0.89	-0.41	-0.64	-0.1
ykfB	-1.08	-0.73	-0.34	-1.54	0
ykfF	0.405	-0.16	-0.15	1.306	0.905
yafX	0.27	-0.73	-1.46	1.99	1.35
ykfG	0.988	0.007	-0.48	1.709	1.168
ykfH	-0.14	0.483	-0.32	2.153	0.908
yafW	0.759	0.259	0.347	2.243	1.241
ykfI	0.532	0.16	0.499	3.25	0.873
alpA	0.164	0.931	-0.4	0.237	2.038
yfjI	0.992	1.119	-0.59	2.704	1.972
yfjJ	2.99	3.07	0.58	3.05	2
yhjV	2.42	1.78	0.86	0.7	0.94
cysQ	-0.67	0.282	0.326	-0.5	-0.81
ytfI	2.59	3.16	2.26	3.26	1
yibA	1.052	1.31	0.371	2.03	0.44
yibV	4.11	4.21	0.33	5.82	2.75
ycfJ	2.97	2.83	-0.47	0.5	-0.03
yegQ	2.25	1.18	-1.31	1.44	-0.38
mdtA	1.05	0.54	1.62	3.6	2.15
mdtE	1.21	1.23	0.24	4.16	3.51
mdtF	1.32	1.32	0.69	2.94	2.73
rfaQ	0.76	0.6	-0.34	-0.03	-0.19
rfaG	1.5	1.2	0.11	0.44	0.24
rfaZ	0.93	0.86	0.81	0.87	0.19
rfaL	1.02	0.34	-1.19	0.49	-0.47
rfaF	0.76	0.6	-0.34	-0.03	-0.19
yafQ	1.29	0.86	0.08	0.4	0.58
dinJ	-0.32	-0.09	-0.83	-0.94	-0.21

Gene Name	*nusG* G146D	*nusG* V160N	*nusG* L158Q	*rho* N340S	BCM
yeeU	-0.73	-0.93	-0.3	-0.32	-0.09
yeeV	-1.38	-1.54	-0.46	-0.78	-1.12
yeeR	-0.25	-0.34	-0.77	-1.63	-1.35
yeeS	-0.09	-0.17	-0.66	-1.53	-0.55
rfaD	0.57	0.72	-0.14	-0.72	-1.48
rsd	-1.07	-0.33	0.241	-0.28	-0.33
yagN	-0.28	0.307	0.979	0.133	0.926
glyU	0.76	0.8	0.26	-0.14	-0.51
ndh	2.03	1.57	-1.64	-1.05	-2.35
gltD	-0.34	0.22	0.3	0.99	-0.2
ydfU	2.36	1.95	1.4	2.77	1.76
ydeQ	2.94	2.86	0.82	3.63	4.85
cspH	0.27	-0.08	2.13	-0.95	-1.54
selC	-0.13	-0.64	-0.76	-0.23	-0.61
ydeP	1.33	1.71	-0.83	0.8	0.56
pgaA	1.43	0.95	2.72	3.03	4.22
ydhQ	-1.12	-1.13	-1.03	-1.5	-2.71
topA	-0.37	-0.67	-0.75	0.17	-0.59
osmY	0.15	0.21	-1.19	2.43	1.88
dmsA	1.48	0.99	0.32	0.96	-0.15
ptsG	0.71	0.66	-0.15	-0.02	-0.72
topB	0.22	-0.19	0.1	-0.56	-0.73
yeiT	0.03	0.3	0.29	1.74	3.04
gntX	0.1	-0.08	0.11	0.9	-0.27
corA	1.19	0.98	0.25	1.59	0.99
sodA	-0.39	-0.19	0.14	-1.39	-1.48
yiiS	0.01	0.51	0.61	0.76	0.36
uvrB	0.81	1.06	-0.09	-0.38	-0.04

Figure S1: Gene expression levels of selected genes in the Rho mutants relative to the WT strain obtained from the microarray experiments reported in Shashni et al., 2014. . Genes denoted in pink shade describes their expression levels in the presence of Bicyclomicin (BCM) relative to its absence, and are obtained from Peters et al., 2012. Genes depicted in the blue were tested in *in vitro* Rho-dependent transcription termination described in Nadiras et al., 2018. Genes in shade of green are taken from the 3’ end mapping described in Dar and Sorek, 2018.

Rho structure and NusG structures with the location of the mutants used in the study

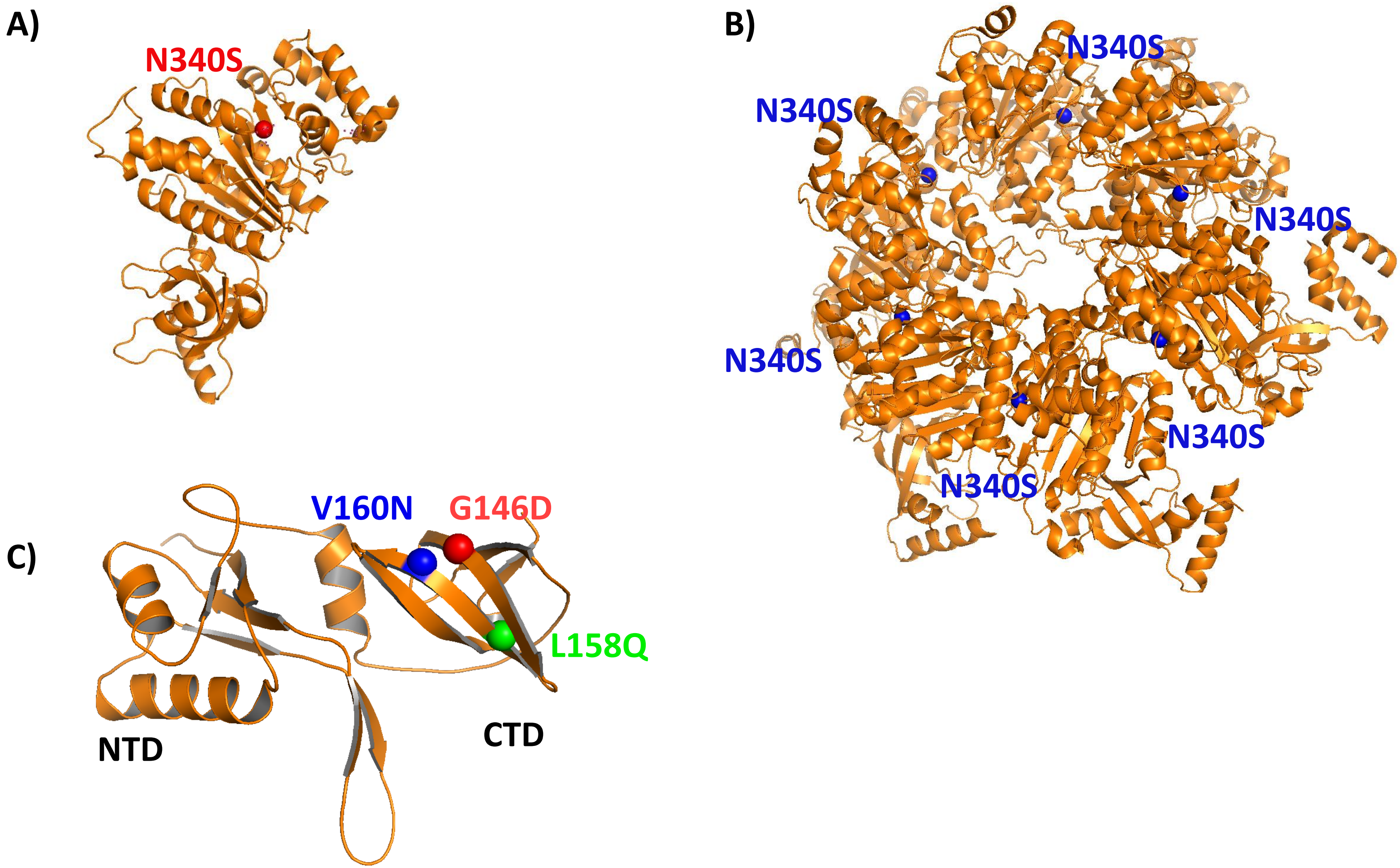

Figure S2. A) Rho monomer showing the position of N340S mutation PDB id: 3ICE). B) Rho hexamer showing the position of N340S mutation. N340S mutation is located in the central channel at the secondary RNA binding site of Rho hexamer is located. C) Location of NusG mutation in the CTD of NusG (V160N (blue), G146D (red), L158Q (green) (PDB ID: 2JVV).

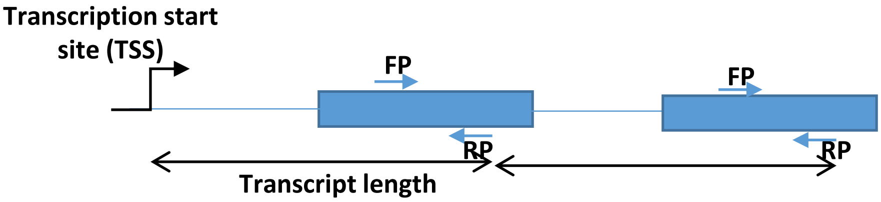

Gene Name	Reverse probe (RP) distance from TSS	Gene Name	Reverse probe (RP) distance from TSS	Gene Name	Reverse probe (RP) distance from TSS
gltD	5914	*yhjV*	861	*ydiL*	504
yeeS	4316	*cadB*	834	*rsd*	499
gatD	4008	*gfcA*	830	*rfaH*	484
topB	3204	*mdtA*	829	*yfjJ*	480
ydeT	3056	*waaQ*	811	*gntX*	461
Trac	1983	*ytfI*	807	*dmsA*	432
sbp	1900	*yafZ*	804	*sodA*	431
thiC	1861	*acrA*	799	*ykfI*	424
yagM	1727	*corA*	781	*yeiT*	416
waaZ	1633	*ygeQ*	763	*selC*	402
pgaA	1501	*cadC*	762	*sugE*	370
katE	1440	*topA*	755	*dinJ*	354
ydeN	1418	*ptsG*	749	*puuD*	347
ndh	1329	*sanA*	708	*rcn*	333
tnaA	1292	*Trpt*	661	*cspH*	316
ydfU	1200	*yfhR*	658	*thrW*	258
ydeQ	1183	*rfaD*	647	*yiiS*	250
dppA	1050	*thiM*	640	*ldrD*	240
TR1	1011	*yafQ*	633	*ykfG*	128
tolC	948	*osmY*	629	*istR*	123
pspB	943	*yagL*	558	*hcp*	121
yfjI	929	*ycfJ*	524	*bdm*	102
ydhQ	904	*uvrB*	513	*sokC*	91

Figure S3: Length of the RNA transcript of the genes, expression levels of which were measured in figure 2A by RT-qPCR

A)

Descriptor	Calculation
Length of the longest C>G bubble	L1
Cumulated length of all C>G bubbles	L1+L2
Density of C>G bubbles (in length)	(L1+L2)/L_t
Area of the longest C>G bubble	S1
Cumulated area of all C>G bubbles	S1+S2
Density of C>G bubbles (in area)	(S1+S2)/L_t
Maximal %C in longest C>G bubble	%CmaxBub
Minimal %G in longest C>G bubble	%GminBub
Maximal %C-%G in longest C>G bubble	(C-G)max%Bub
Average %C-%G in longest C>G bubble	(C-G)av%Bub
Number of [(YC)N9→13]1 motifs (aka YC dimers) in longest C>G bubble	2 (Y1 and Y2)
Number of [(YC)N9→13]2 motifs in longest C>G bubble	1 (Y3)
Number of [(YC)N9→13]3 motifs in longest C>G bubble	None
Density of [(YC)N9→13]1 motifs (aka %YC)	(Total number [(YC)N9→13]1)/ L_t i.e. (2/Lt)

B)

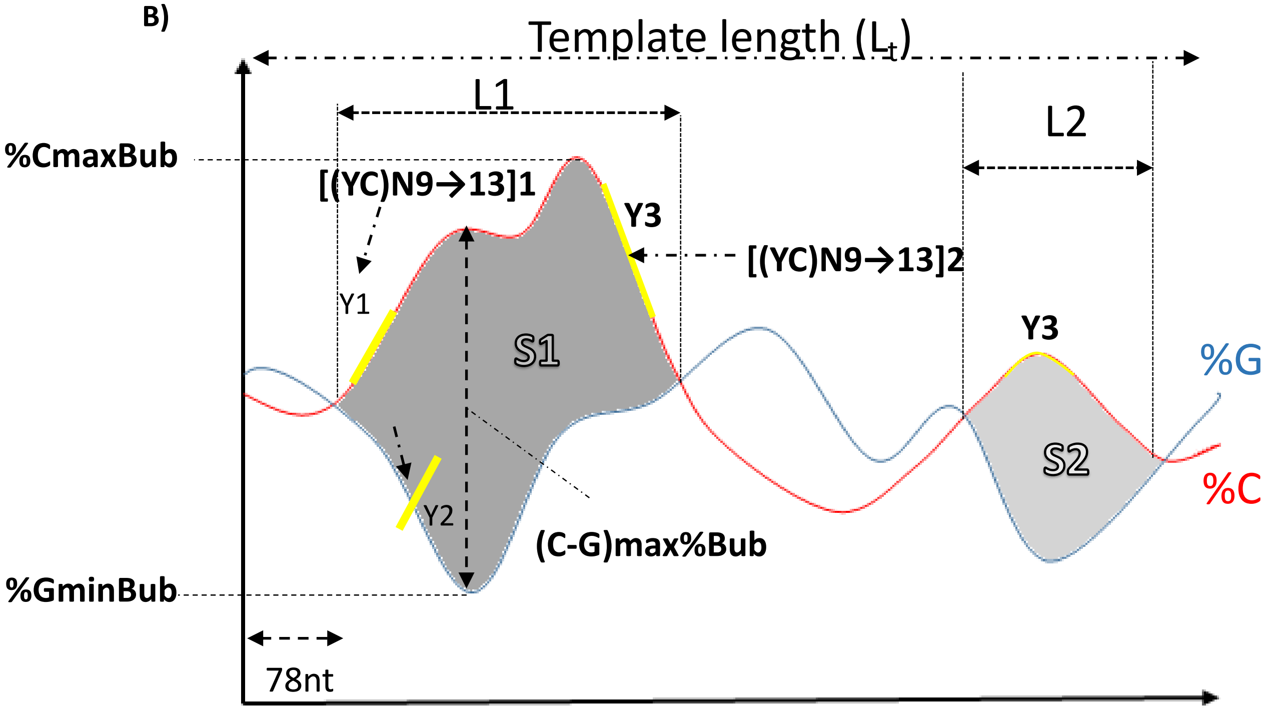

C)

Length of the longest C > G bubble calculates the length of the longest bubble (L1).
Cumulated length of all C > G bubbles calculates the sum of all C > G bubbles length (L1+L2) within the sequence of 78 nt window.
Density of C > G bubbles (along the length) describes the fraction of cumulated length of all C > G bubbles relative to the total length of the sequence (Lt) ((L1+L2) / Lt).
Area of a bubble (Si in Figure S6) was determined by summing up %C - %G values over the length of the bubble (area are thus expressed in % x bp).
Area of longest C > G bubble describes the area that comes under the longest C > G bubble (S1).
Cumulated area of all C > G bubbles describes the sum of the area under all C > G bubbles (S1+S2).
Area density of C > G bubbles describes the fraction of the cumulated area of all C > G bubbles along the length of the sequence under study (S1+S2)/Lt).
Maximal %C in longest C > G bubble describes the maximum %C value, among all the 78nt sequence windows within the longest C>G bubble.
Maximum %C - %G in longest C > G bubble describes the point of largest difference between the %C and %G ((C-G)max% Bub).
Average %C - %G in longest C > G bubble describes the average of the difference between %C and %G at each point within a C > G bubble (C-G) av % Bub).
Number of [(YC)N9→13] 1/ 2/ 3 motifs in longest C > G bubble counts the numbers of YC dimer motifs, present inside the longest C > G bubble.

- When the dimer motifs are repeated distances between the two consecutive motifs will be 9 to 13 bases. Number of [(YC)N9→13] 1 counts all YC dimers.
- [(YC)N9→13] 2 counts the occurrence of two consecutive YC dimers.
- [(YC)N9→13] 3 counts the occurrence of three consecutive YC dimers.

Density of [(YC)N9→13]1 motif (%YC) calculates the fraction of the number of YC dimers within the length of the sequence under study ((Number of [(YC)N9→13]1)/L_t).

Figure S4. A) Definitions of the descriptors and representation of calculation of the descriptor values. B) Graphical representation of the C>G plots. C) Description of the descriptors used in the study.

(The definitions of the descriptors were taken from Naridas *et.al.,* 2018)

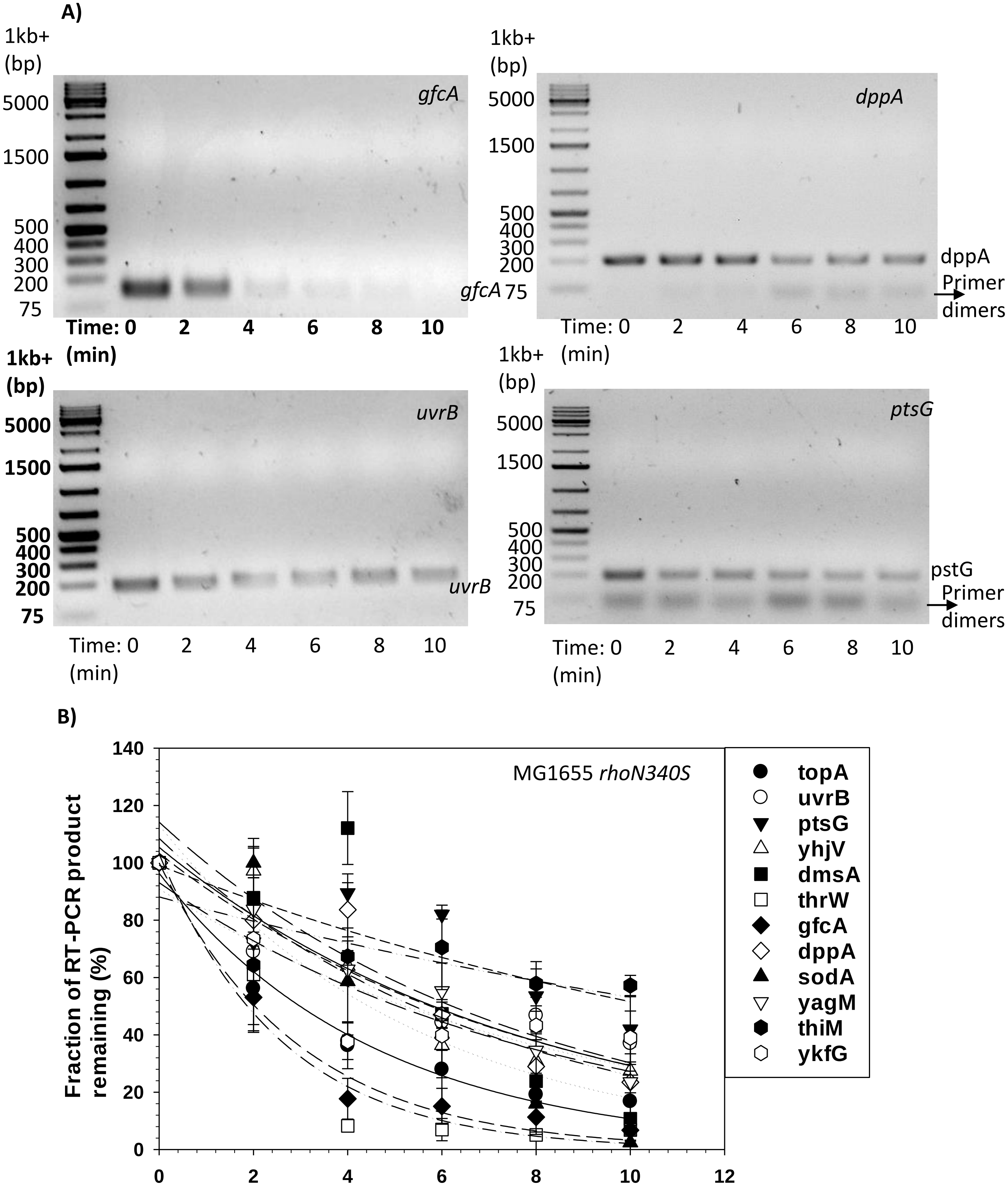

Figure S5: A) Time dependent RT-PCR products (cDNA) of the indicated genes obtained from the RNA preparations from MG1655 *rho*N340S at the indicated time-points. The cDNA band and that from the primer dimers are indicated together with the DNA molecular weight markers. B) Fractions of RT-PCR products obtained from indicated genes against time. The points were fitted to exponential decay equation to calculate the rate of decay and half-lives of the products.

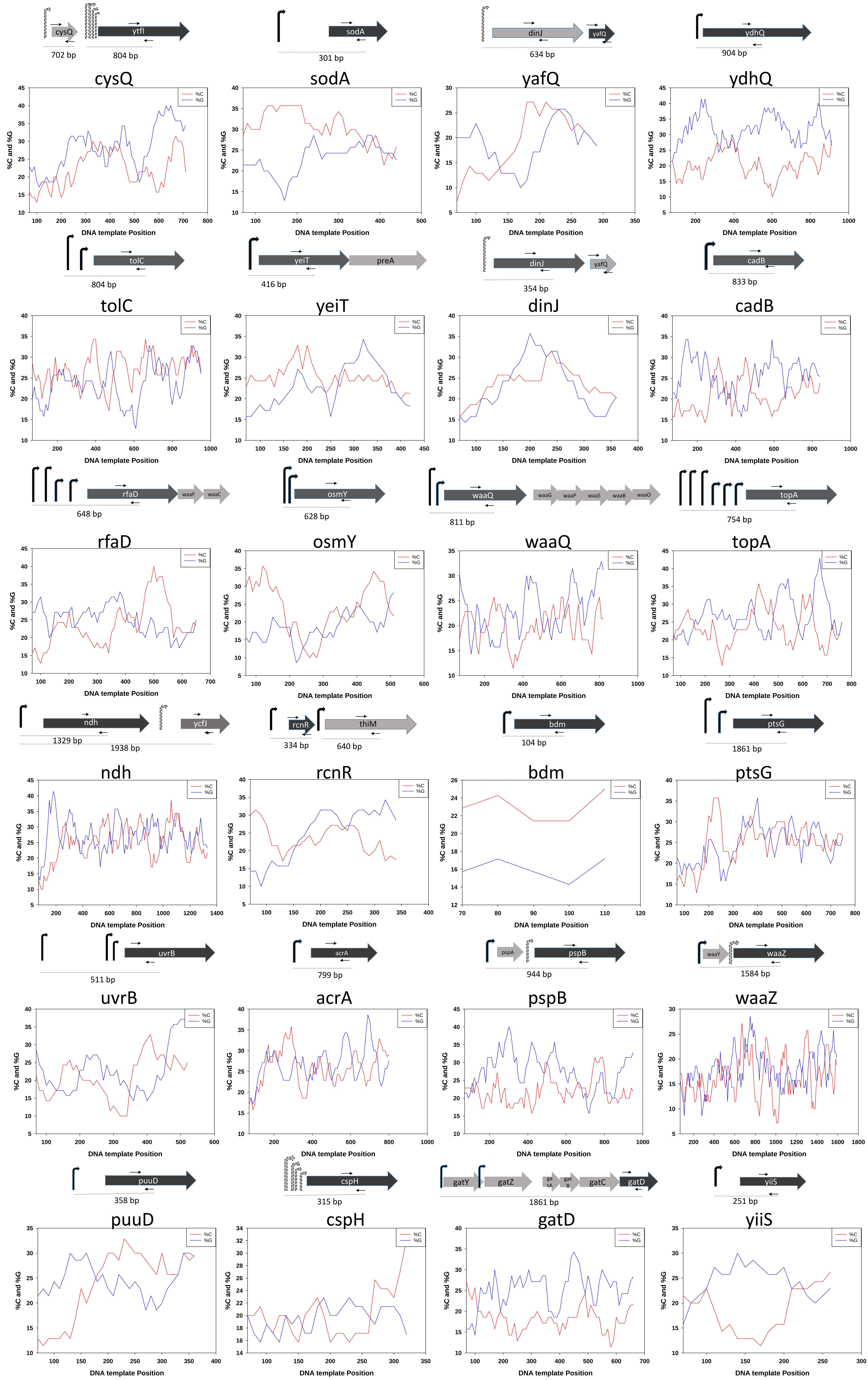

Figure S6: C > G bubble plots for the untranslated sequences of the all the genes used in this study.

Figure S6 part A

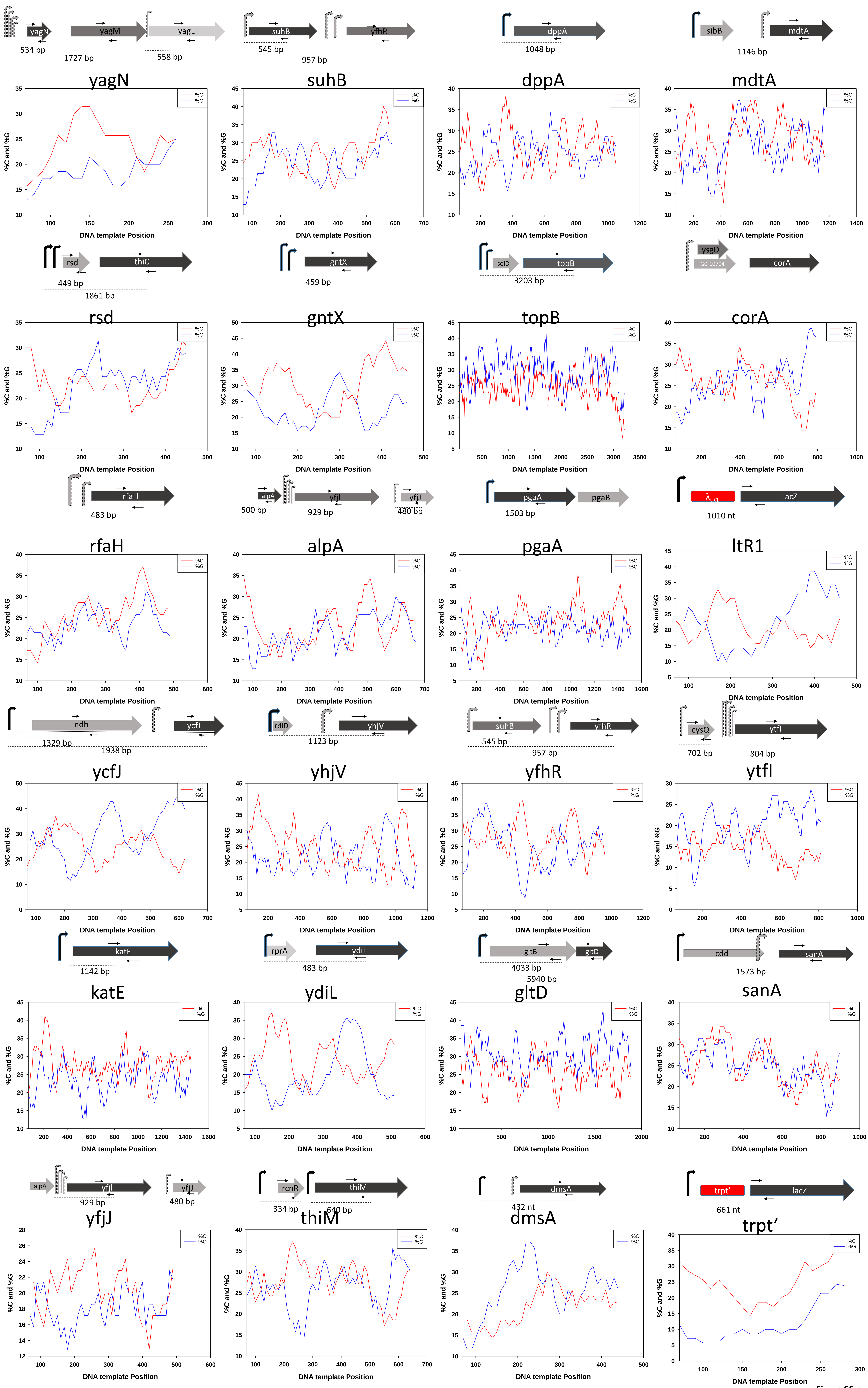

Figure S6 part B

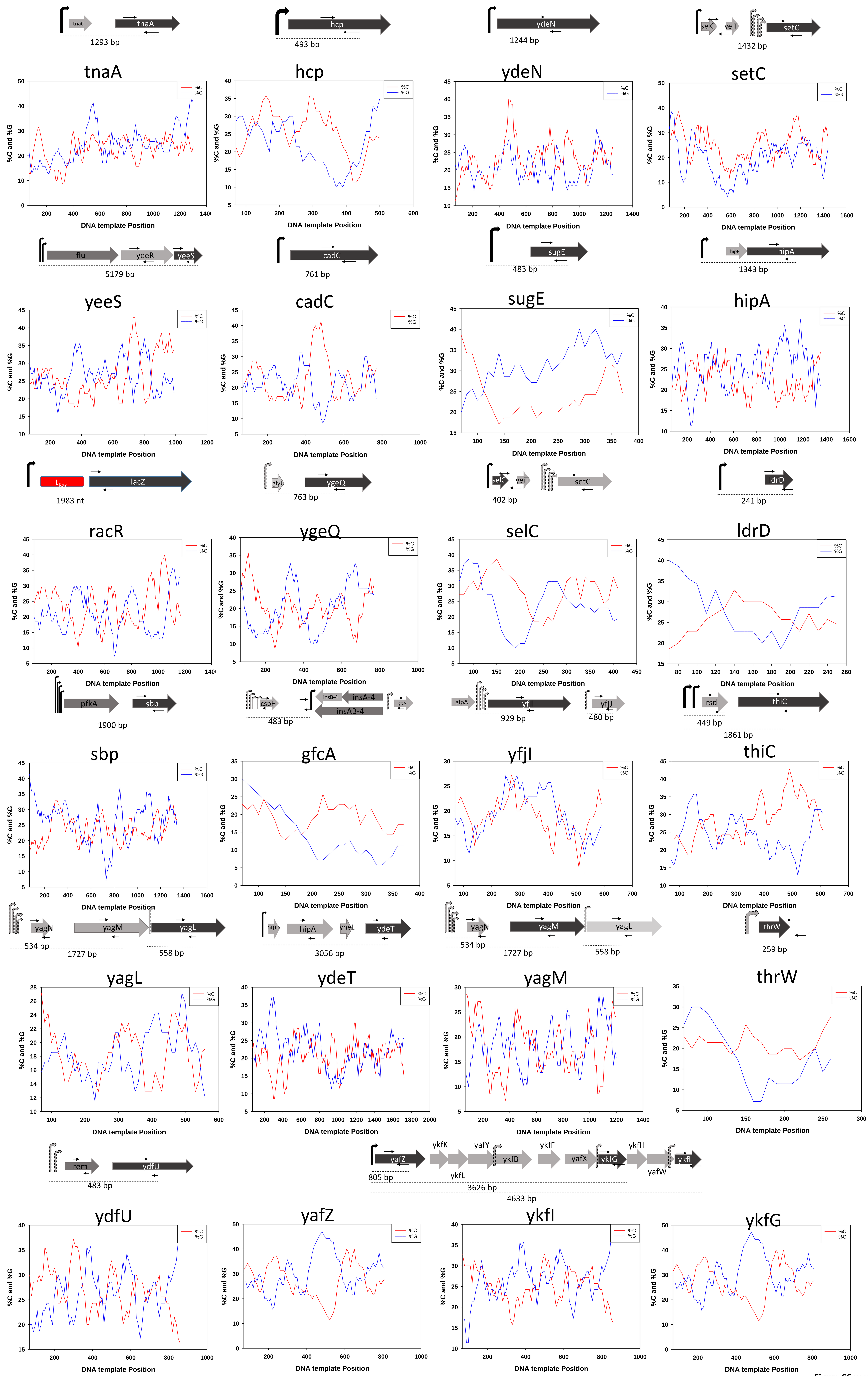

Figure S6 part C

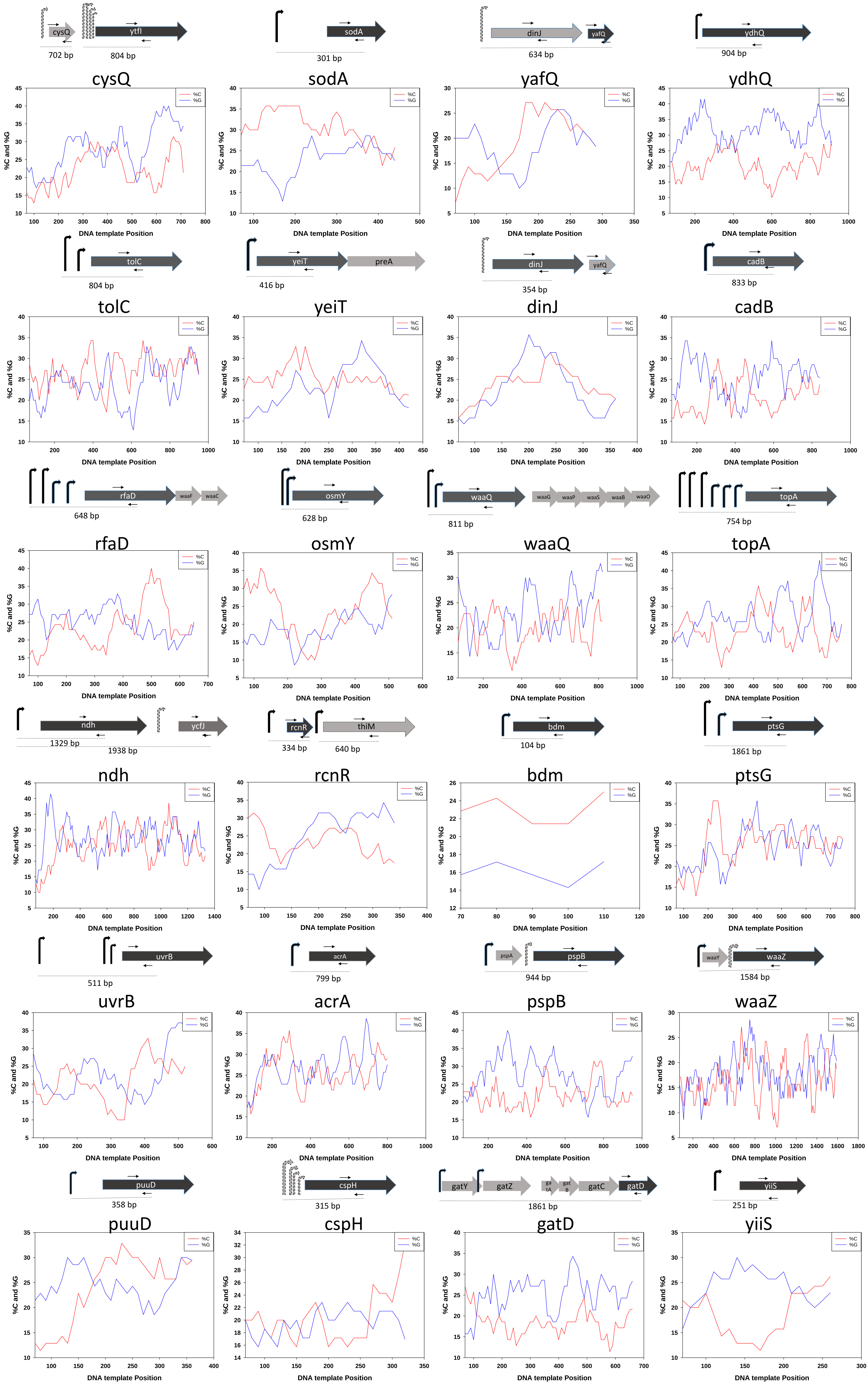

Figure S6: C > G bubble plots for the untranslated sequences of the all the genes used in this study.

Figure S6 part A

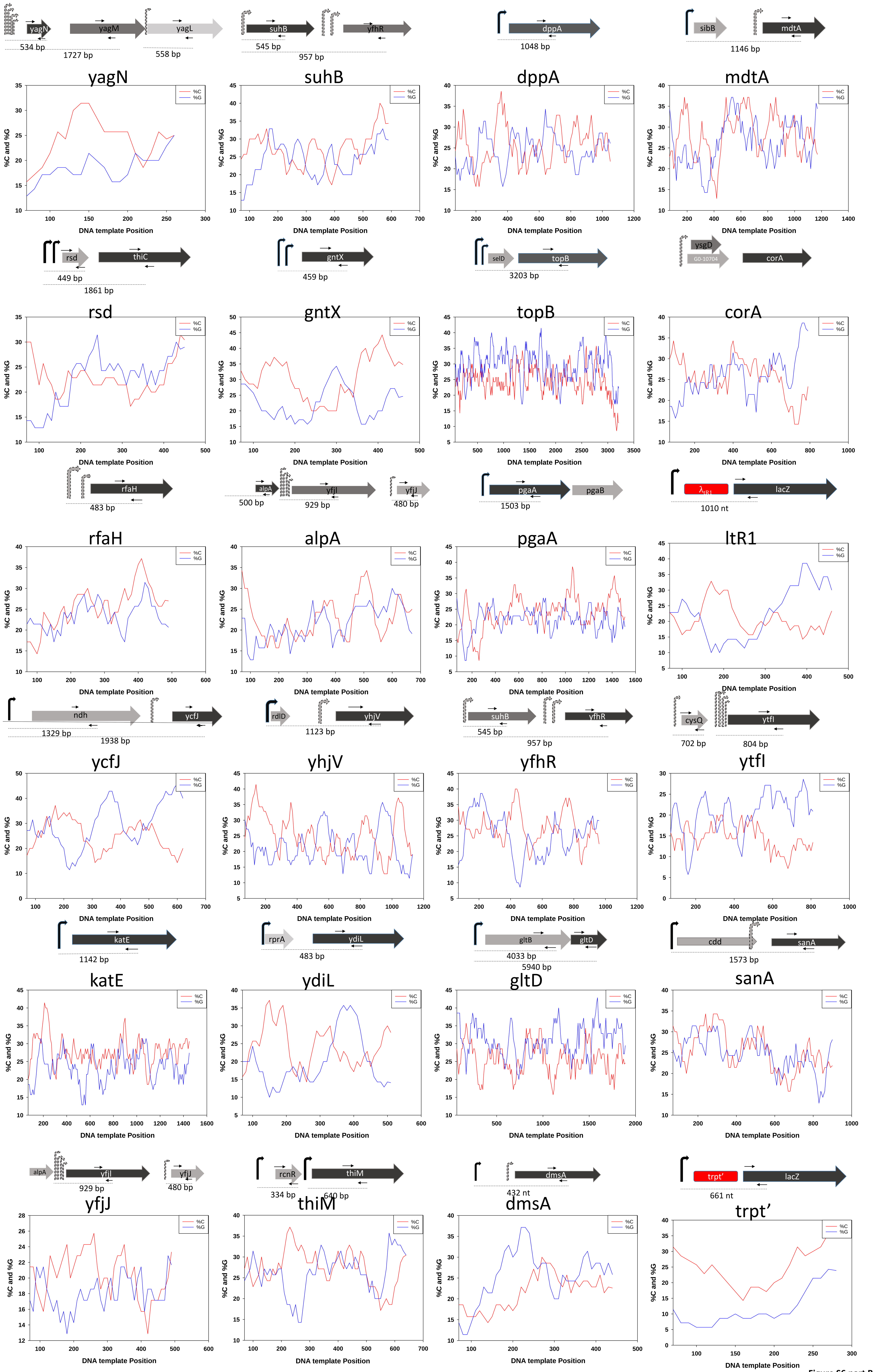

Figure S6 part B

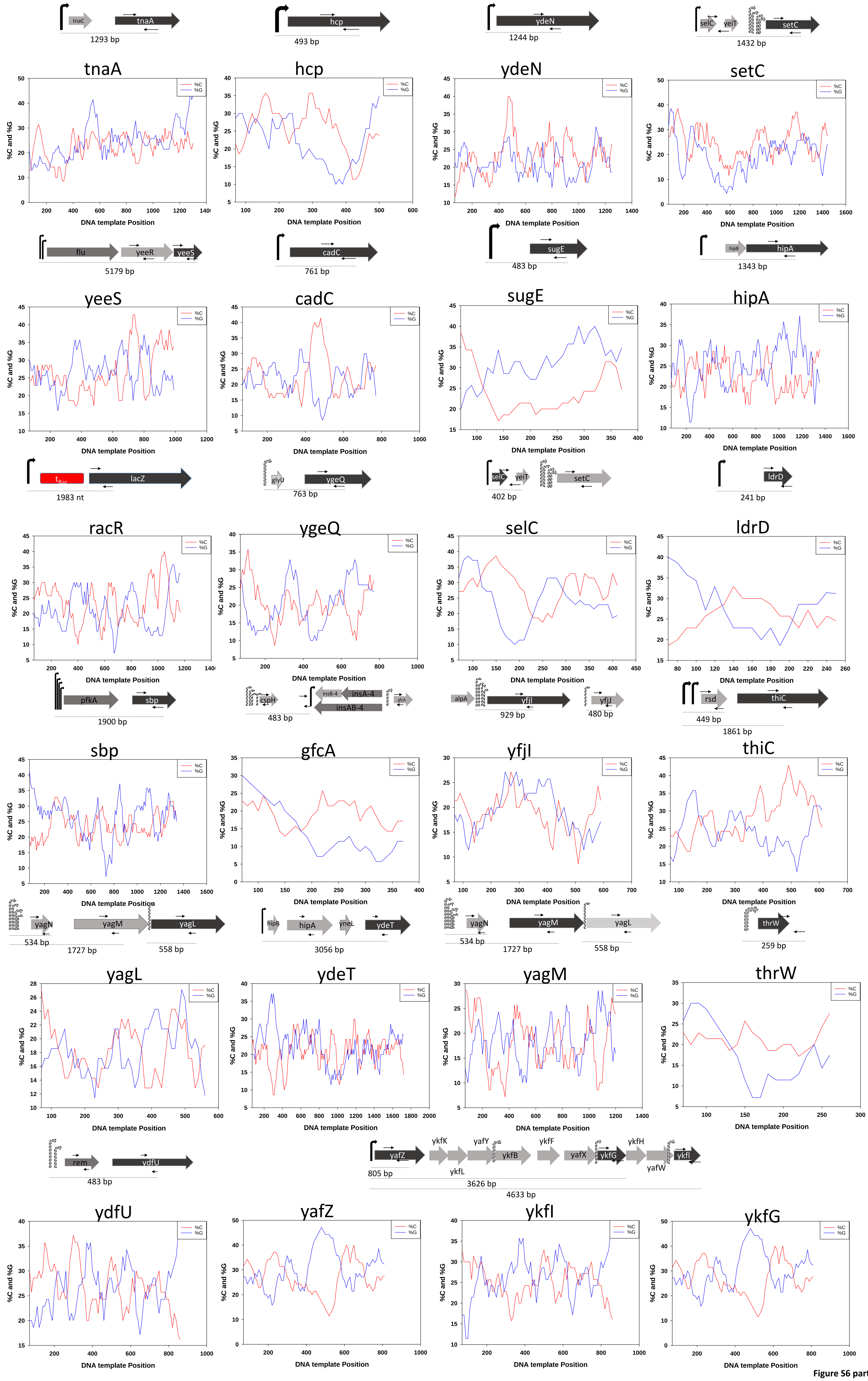

Figure S6 part C

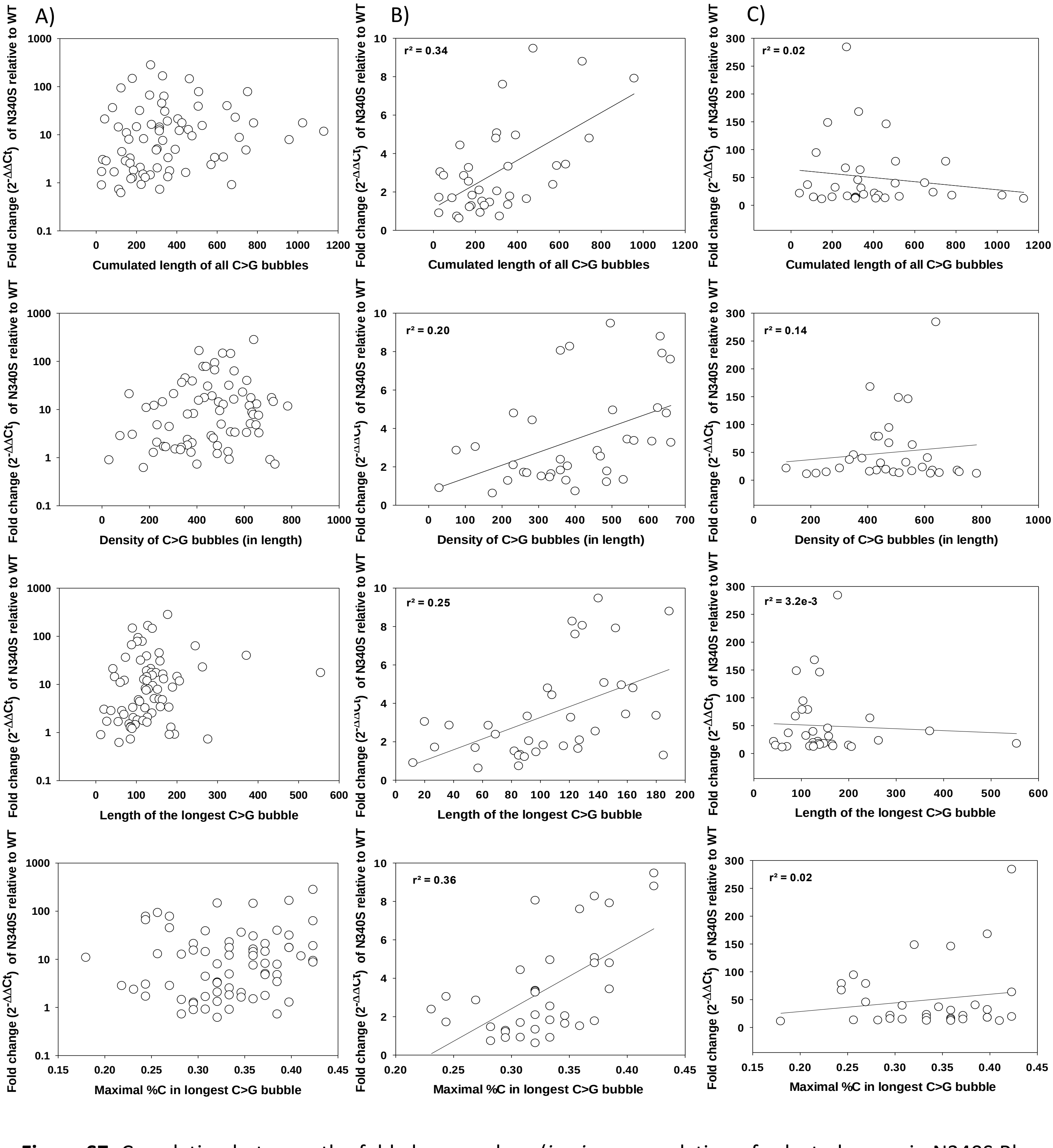

Figure S7. Correlation between the fold-change values (*in vivo* upregulation of selected genes in N340S Rho relative to WT, from figure 2A) and the indicated descriptors. Descriptors are: Cumulated length of all C>G bubbles, Density of C>G bubbles (in length), Length of the longest C>G bubble and Maximal %C in longest C>G bubble are depicted in each row respectively. **A) S**cattered plots of all ranges of fold change (log scale) plotted against the descriptor values. **B**) The terminators with <10-fold change (0-10 fold upregulation) plotted against the descriptor values and fitted to a straight line equation. Three most disruptive outliers had been eliminated in each case: Cumulated area of all C>G bubble: λt_{R1}, *tolC* and *soda;* Density of C>G bubble (in length): *yeiT*, *tolC* and *soda;* Length of longest C>G bubble: *yeiT*, *tolC* and *soda;* Maximal %C in longest C>G bubble: *cspH*, *rfaD* and *sodA*. **C)** The terminators having > 10-fold change (>10 fold upregulation) plotted against the descriptor values.

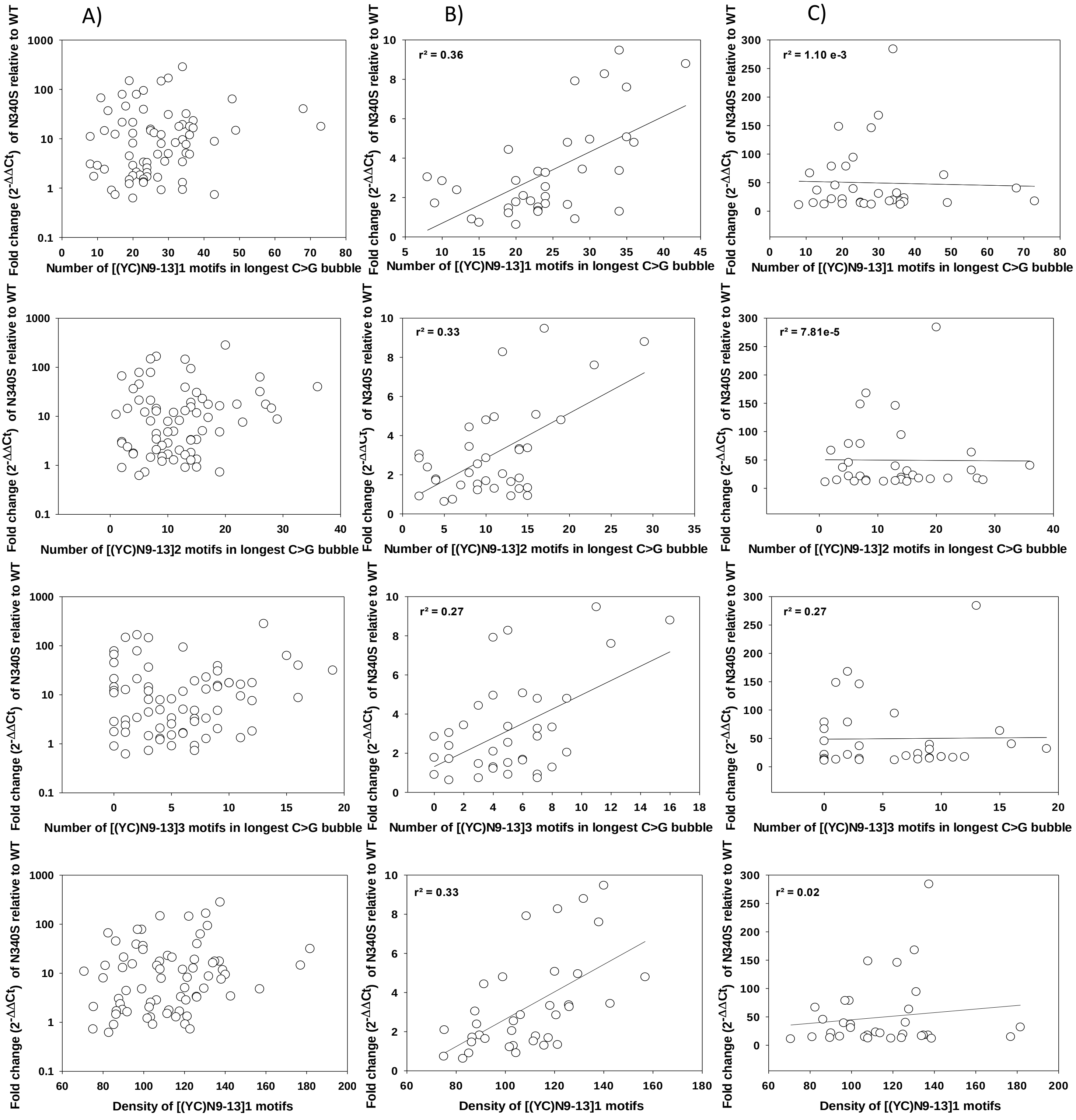

Figure S8. Correlation between the fold-change values (*in vivo* upregulation of selected genes in N340S Rho relative to WT, from figure 2A) and the indicated descriptors. Descriptors related to YC repeats: number of [(YC)N9→13]1 motifs in longest C>G bubble, number of [(YC)N9→13]2 motifs in longest C>G bubble, number of [(YC)N9→13]3 motifs in longest C>G bubble and density of [(YC)N9→13]1 motifs are used. **A)** Scattered plots of fold change values of all the terminator (log scale) plotted against the descriptor values. **B)** The fold change values< 10-fold (fold upregulation) plotted against the descriptor values. Three most disruptive outliers were eliminated in each case. Number of [(YC)N9→13]1 motifs in longest C>G bubble: *λ*t_{R1},*yeiT* and *sodA.* Number of [(YC)N9→13]2 motifs in longest C>G bubble: *λ*t_{R1}, *pgaA* and *sodA*, Number of [(YC)N9→13]3 motifs in longest C>G bubble: λt_{R1}, *uvrB* and *sodA*. Density of [(YC)N9→13]1 motifs (aka %YC): λt_{R1}, *yeiT* and *sodA*. **C)** The fold change values >10 fold (upregulation) plotted against the descriptor values.

A)

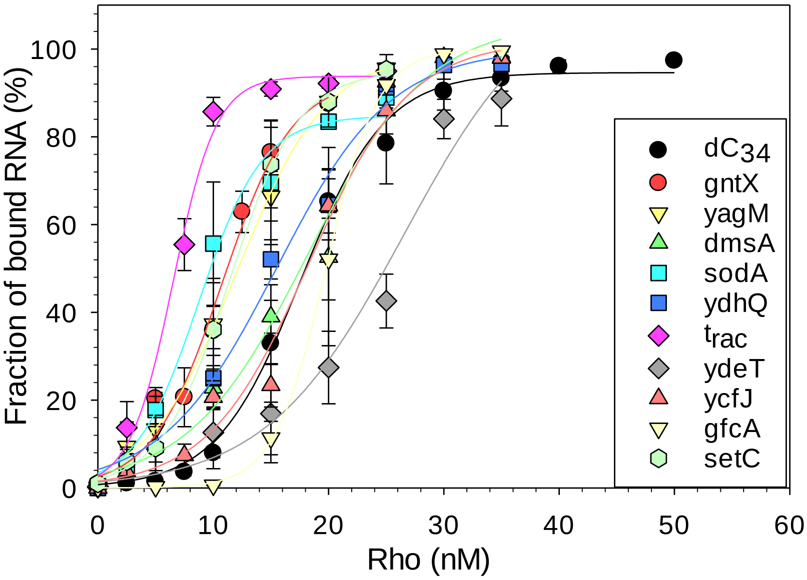

B)

Terminator	Kd	Fold change
t_{rac}	3.08 ± 0.97	23.07 ± 4.96
sodA	9.99 ± 2.83	0.73 ± 0.07
setC	11.52 ± 1.80	31.85 ± 1.87
yagM	11.80 ± 0.90	78.60 ± 14.86
dmsA	16.15 ± 0.54	14.48 ± 4.12
ydeT	16.33 ± 1.35	78.59 ± 31.25
ydhQ	16.45 ± 2.52	0.90 ± 0.22
ycfJ	18.47 ± 0.58	8.27 ± 3.48
gfcA	19.89 ± 0.60	40.13 ± 10.14
gntX	23.18 ± 0.51	4.79 ± 0.20
dC_{34}	18.02 ± 2.05	-

C)

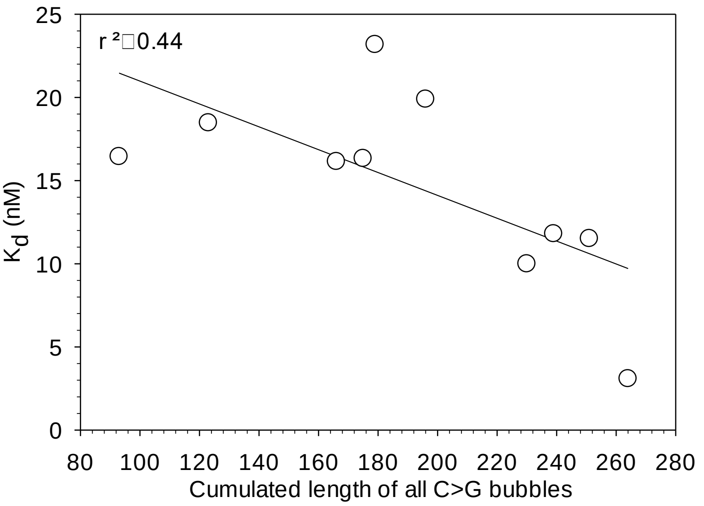

D)

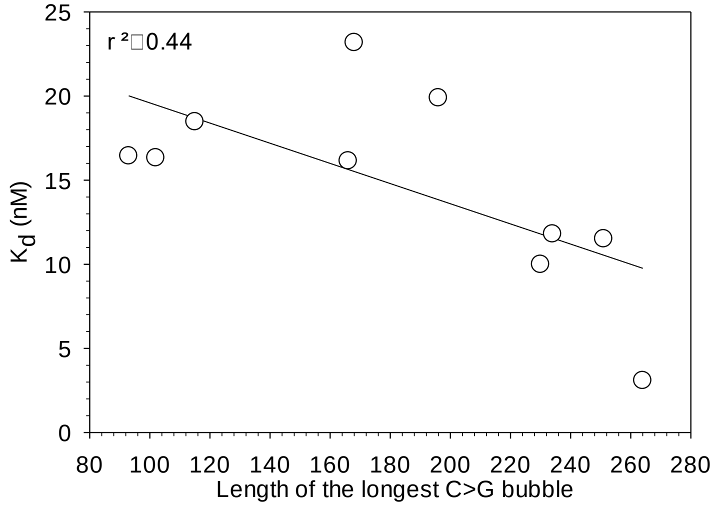

E)

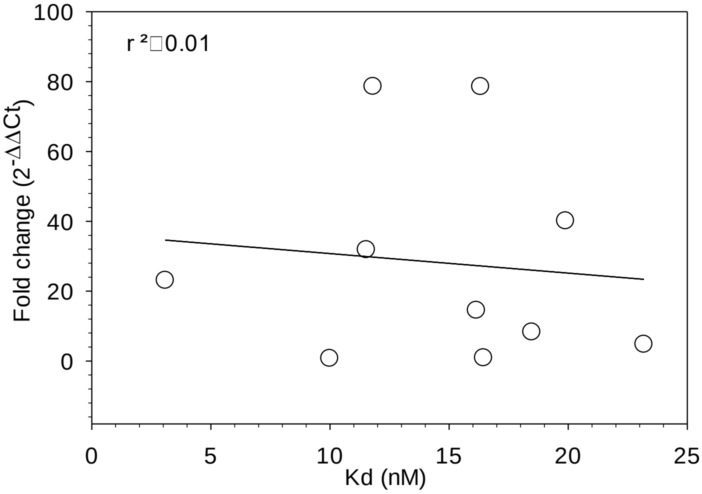

Figure S9. **A)** Binding isotherms of Rho obtained for RNA templates made from the untranslated regions of the indicated genes by EMSA. poydC_{34} is a 34-mer synthetic DNA. **B)** All the plots are fitted to sigmoidal equation of the form y = a/(1+exp(-(x-x0)/b)) to obtain the dissociation constant, K_d (b in the equation) that are shown in a table along with the measured *in vivo* fold change of N340S Rho (relative to WT obtained from figure 2A). **C)** and **D)** Kd s are plotted against the indicated descriptor valuese to obtain the co-relation coefficient r^2. **E)** Fold change values are plotted against the Kd values. Fold change values are obtained from figure 2.

F)

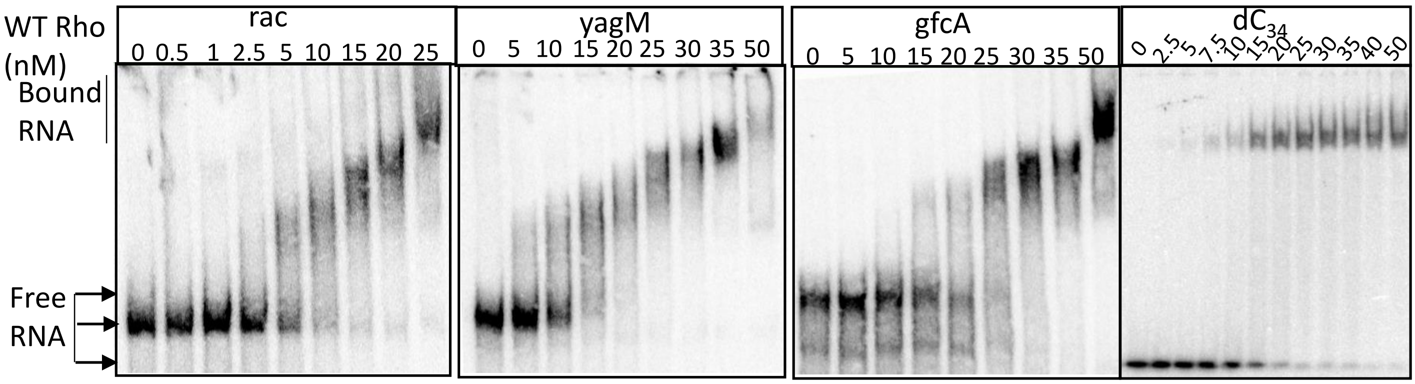

G)

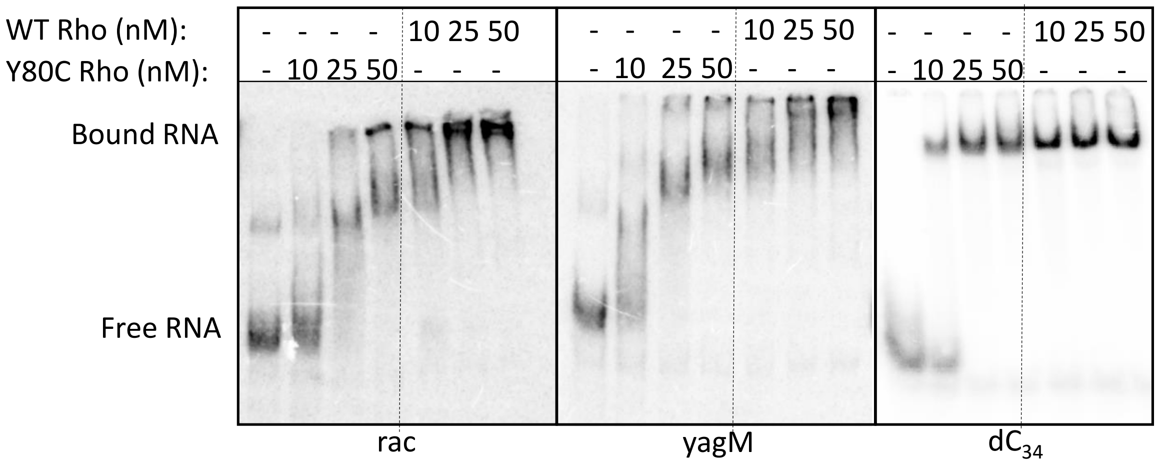

Figure S9. **F)** Representative autoradiograms of the gel-shift assays with indicated RNAs (rac, YagM, gfC and dC_{34}) with WT Rho to calculate the K_d of Rho on the RNA templates. **G)** Representative autoradiograms showing the comparison of RNA binding of WT and the Y80C mutant Rho to the indicated RNAs. RNA templates are derived from the Rho-terminator regions of *rac, yagM, gfC.* dC_{34} is a synthetic 34 mer polyC RNA having very high affinity for WT RNA [17].

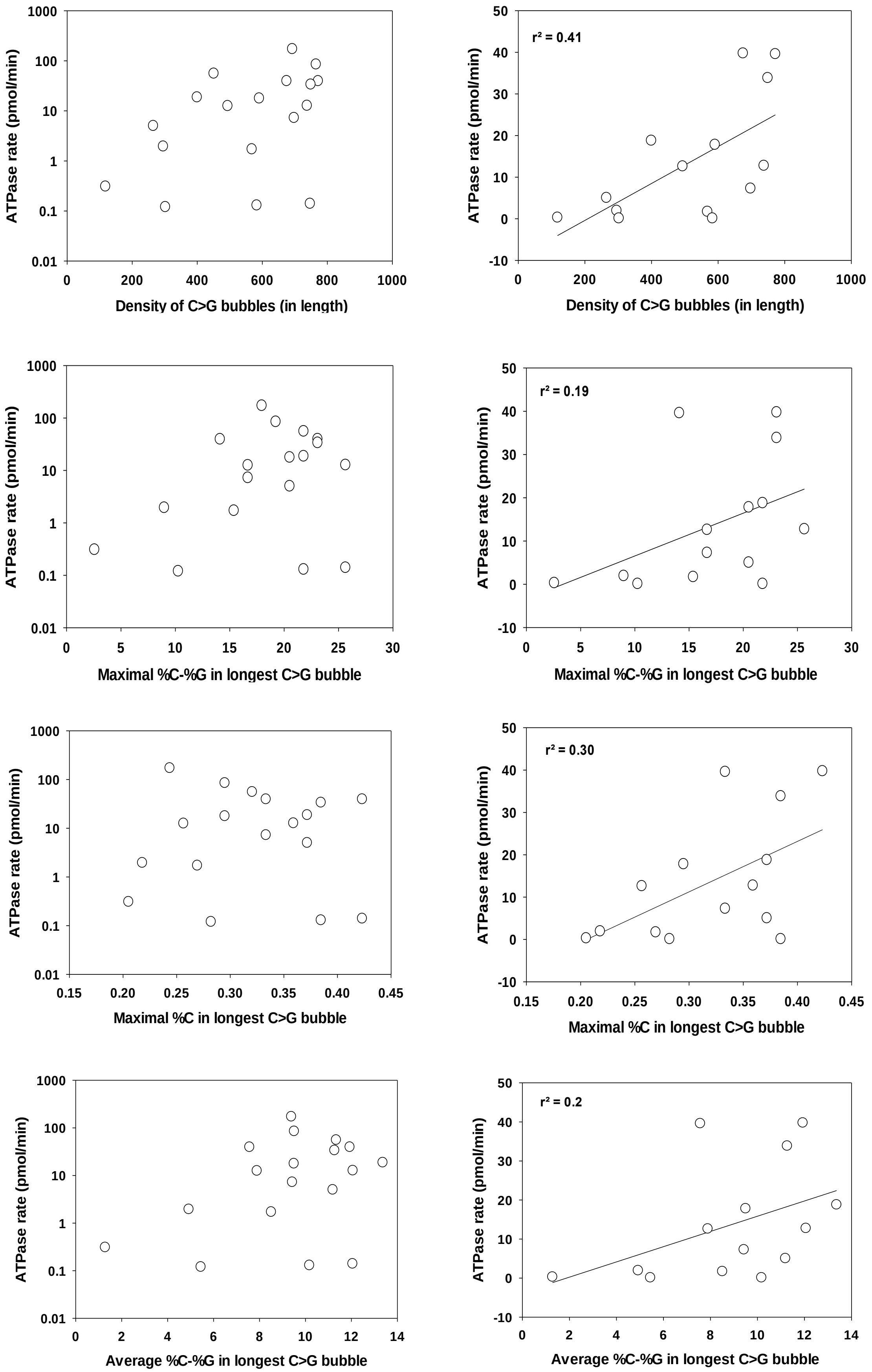

Figure S10. Correlation between the *in vitro* ATPase rates of Rho obtained on different RNA templates and the indicated descriptors. Descriptors are: Density of C>G bubbles (in length), Maximal %C-%G in longest C>G bubble, Maximal %C in longest C>G bubble, Average %C-%G in longest C>G bubble. **A)** Scattered plots of all the data points of the ATPase rates against the indicated descriptor values. **B)** Data points of the ATPase rate (< 50pmol/min) plotted against the indicated descriptor values were fitted to a straight line equation to obtain the correlation co-efficient.

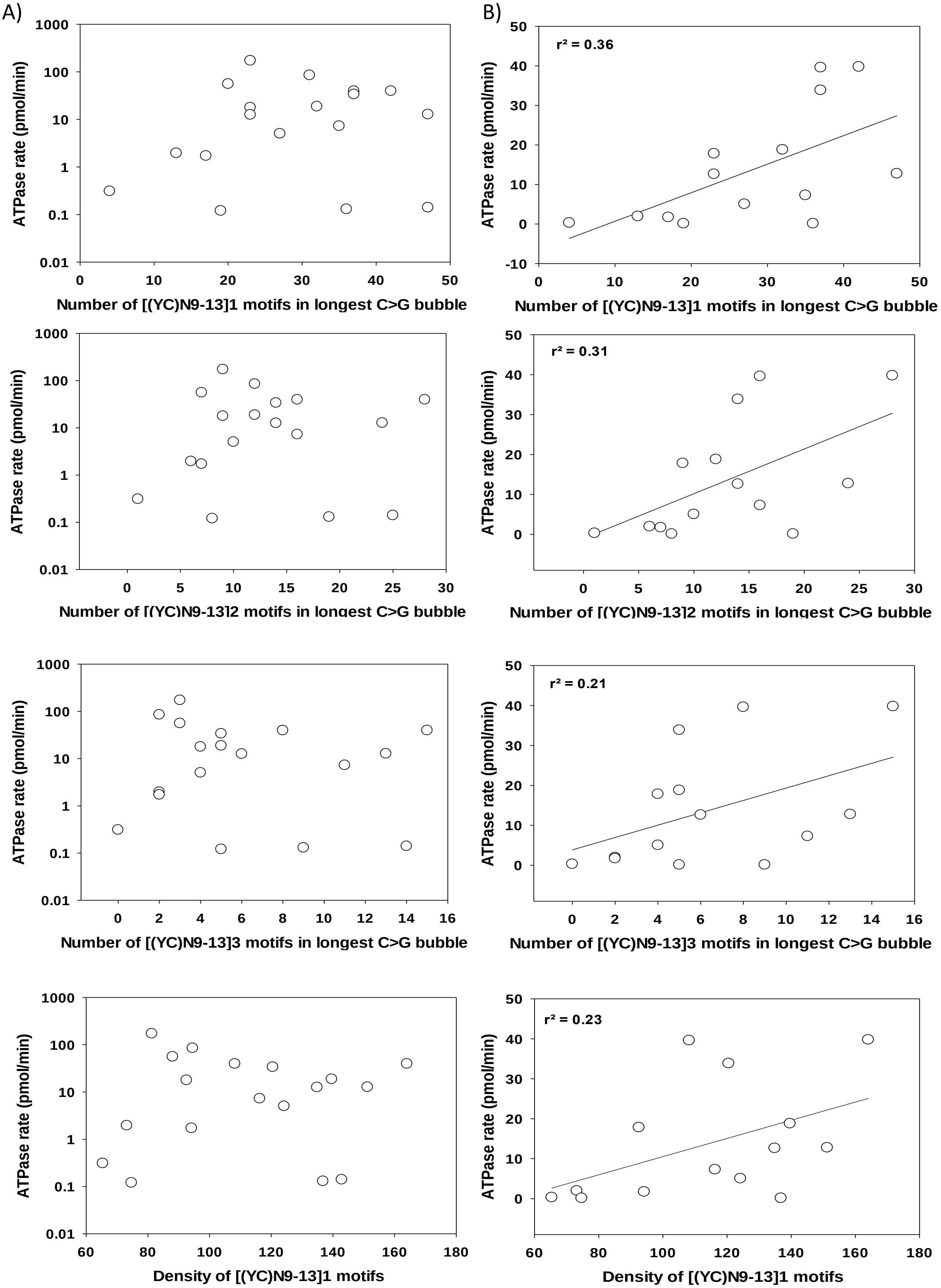

Figure S11. Correlation between the *in vitro* ATPase rates of Rho obtained on different RNA templates and the indicated descriptors. Descriptors are: Number of [(YC)N9-13]1 motifs in longest C>G bubble, Number of [(YC)N9-13]2 motifs in longest C>G bubble, Number of [(YC)N9-13]3 motifs in longest C>G bubble, Density of [(YC)N9-13]1 motifs . **A)** Scattered plots of all the data points of the ATPase rates against the indicated descriptor values. **B)** Data points of the ATPase rate (< 50pmol/min) plotted against the indicated descriptor values were fitted to a straight line equation to obtain the correlation co-efficient.

Gene	Fold change	+/-	No of pause sites after C>G Bubble
ykfG	283.96	29.46	0
yafZ	167.77	33.22	0
ykfI	148.30	0.93	0
ydfU	145.76	13.43	0
thrW	94.11	36.60	0
yagM	78.60	14.86	0
ydeT	78.59	31.25	0
yagL	66.60	7.62	0
thiC	63.39	23.95	4
yfjI	45.31	6.78	0
gfcA	40.13	10.14	0
sbp	39.11	2.34	1
ldrD	36.52	2.44	0
selC	31.85	1.87	0
ygeQ	30.64	5.60	3

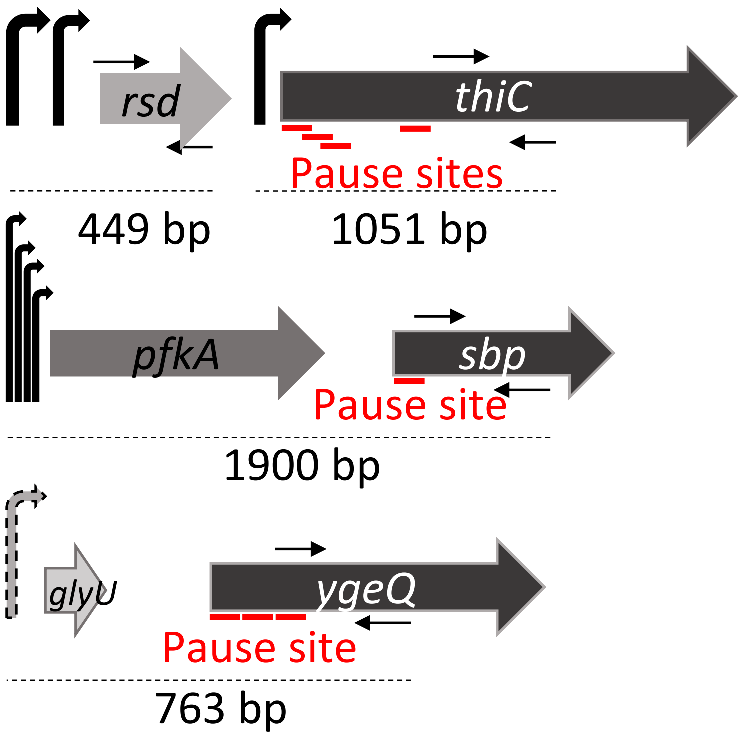

gene	Pause Sequences
	TTCCCAACTCAAAACGCATTTATATCACTG
	GTCTGCAACAAAACTGACCCGCCGCGAACA
	GAATGAGCTATGTCTGCAACAAAACTGACC
thiC	TTTGGAATGAGCTATGTCTGCAACAAAACT
sbp	GGCGATGAACAAGTGGGGCGTAGGGTTAAC
	CAGGGGCAGAAGTGACGAGTGAATCTGTAT
	CGCTGGTATATTTTCGTGCGGCACATGCCA
ygeQ	GAAAGGGAAATCTGCATTAACTCTTCTGCT

Figure S12 . Number of elemental pause sites between the rut sequences and the reverse primer probe (arrows in left to right direction) were determined for those genes that show very high *in vivo* fold change values. The consensus sequence for elemental pause site **GGcataatTG(C/T)GGCcg** was taken from published results (Larson *et.al.,* 2014). Pause sites are indicated us horizontal lines below the genes. Pause site sequences of the three indicated genes are shown in red.

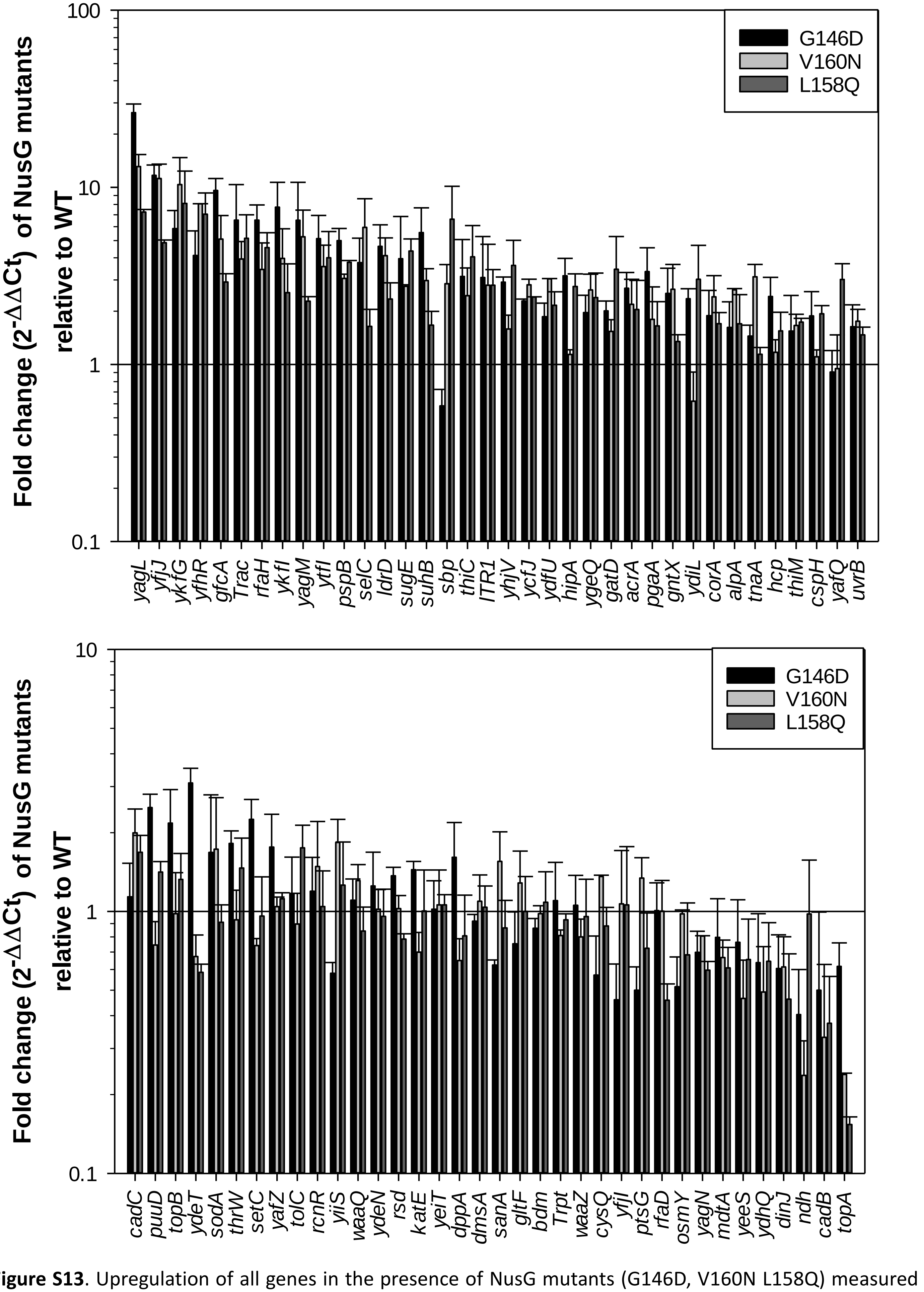

Figure S13. Upregulation of all genes in the presence of NusG mutants (G146D, V160N L158Q) measured by RT-qPCR. **A)** Genes showing higher fold upregulation and **B)** those showing lower fold upregulation.

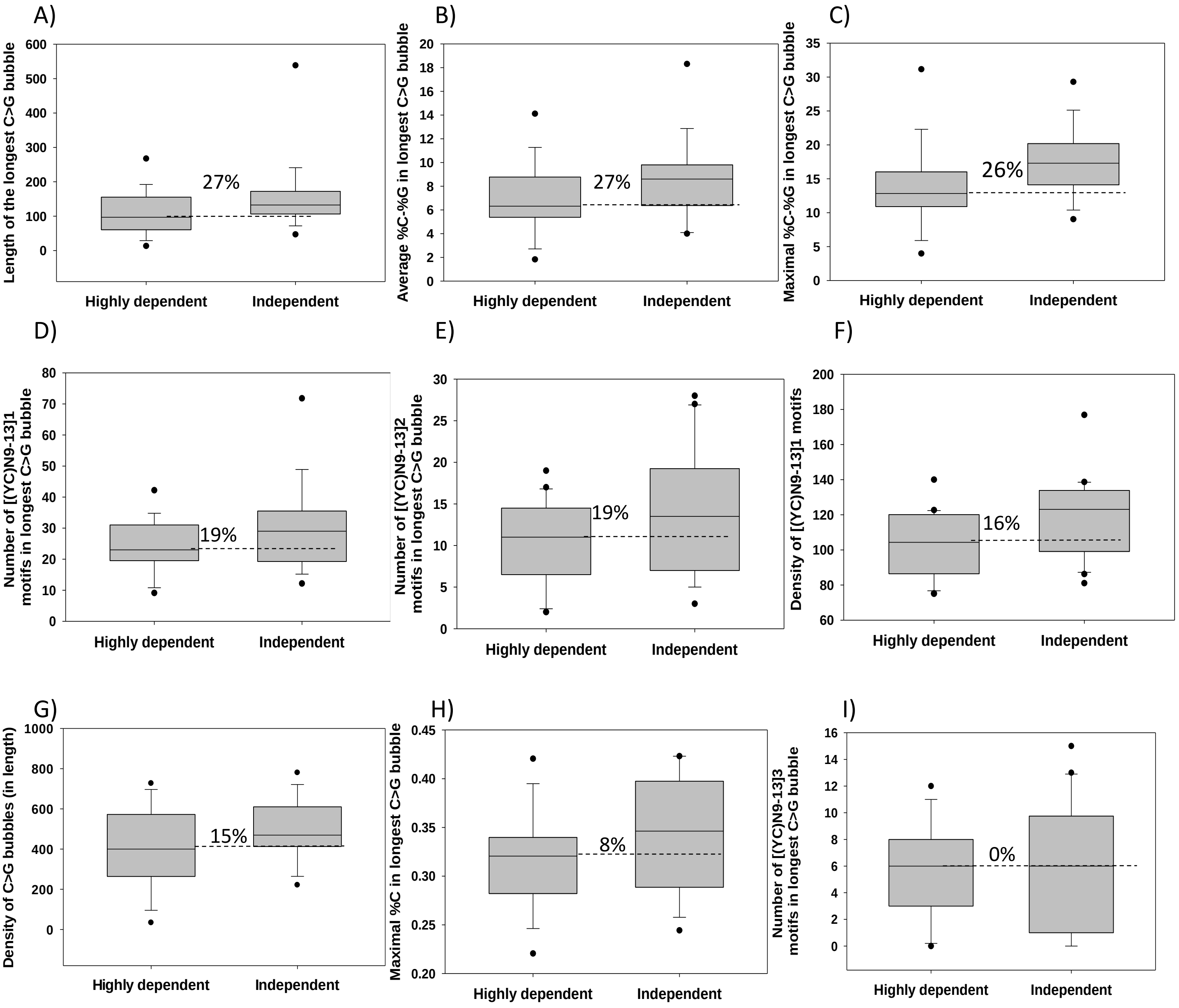

Figure S14. The boxed plots showing the distribution of the indicated descriptor values of the terminators between the ones that are highly NusG dependent and that are independent of NusG (see Figure 6B). The descriptors that are used are: **A)** Length of the longest C>G bubble shows a reduction of 27% in the highly NusG dependent group. **B)** Average %C-%G in longest C>G bubble shows a reduction of 27% in the highly NusG dependent group. **C)** Maximal %C-%G in longest C>G bubble shows a reduction of 26% in the highly NusG dependent group. **D)** Number of [(YC)N9→13]1 in longest C>G bubble shows a reduction of 19% in the highly NusG dependent group. **E)** Number of [(YC)N9→13]2 motifs in longest C>G bubble shows a reduction of 19% in the highly NusG dependent group. **F)** Density of [(YC)N9→13]1 motifs (%YC) shows a reduction of 16% in the highly NusG dependent group. **G)** Density of C > G bubbles (in length) shows a reduction of 15% in the highly NusG dependent group. **H)** Maximal %C in longest C>G bubble shows a reduction of 8% in the highly NusG dependent group. **I)** Number of [(YC)N9→13]3 motifs in longest C>G bubble shows no reduction in the highly NusG dependent group.

Table S1: Oligos used for RT-qPCR and for preparing DNA templates for *in vitro* RNA synthesis (FP and RP are forward and reverse primer, respectively).

Oligo ID	Description	Sequence
RS846	yagM FP for RT-qPCR	CCTTATCCGGTTGTTTGTTG
RS847	yagM RP for RT-qPCR	TTAGGTTCTGCTCTCTTGTG
RS1006	cysQ FP for RT-qPCR	CGATTTTAGGCGTGGTGTATG
RS1007	cysQ RP for RT-qPCR	CCAAGCTGTTGCAGATACTC
RS1308	tolC FP for RT-qPCR	AGCAGCTGCGCCAGATCACC
RS1309	tolC RP for RT-qPCR	CAGAAATCCCGGTAGAAGCCG
RS1312	acrA FP for RT-qPCR	ACACCAAAGTCACCTCTCCG
RS1313	acrA RP for RT-qPCR	GCCGTTCTCTTGTTTCAGCG
RS1325	ykfI FP for RT-qPCR	TGTTACTGACACGTCTGCTGG
RS1326	ykfI RP for RT-qPCR	TGCAGCCCGGAGATAAGGAG
RS1371	mdtA FP for RT-qPCR	GACTGGAGCCGGATTACCG
RS1372	mdtA RP for RT-qPCR	GATCCCAGGCTTCTACCACC
RS1377	ldrD FP for RT-qPCR	ATGACGTTCGCAGAGCTGG
RS1378	ldrD RP for RT-qPCR	CGCTTGTTCAGCCAGTTCAC
RS1421	ytfI FP for RT-qPCR	AACTCCAAATACCAGGAGGTGG
RS1422	ytfI RP for RT-qPCR	TGTTGGCAACCTGCATTAACC
RS1425	yfjJ FP for RT-qPCR	AACTCAACGGAAAGACTGGC
RS1426	yfjJ RP for RT-qPCR	CCAAGTGCACTGTACCAAGC
RS1427	ydeQ FP for RT-qPCR	AAAGCGGGGGAAGTCATTGC
RS1428	ydeQ RP for RT-qPCR	AGGGGTATTTCTGCGCTTCC
RS1429	ycfJ FP for RT-qPCR	CAACCCCAATCAAGGAAACGG
RS1430	ycfJ RP for RT-qPCR	TGGTTACCGGCATATCCACC
RS1431	yagL FP for RT-qPCR	ACATGTTGTGATCGAGCAGG
RS1432	yagL RP for RT-qPCR	TCCTTGAAGCTGGATGGTAGC
RS1466	dppA FP for RT-qPCR	TTTGATGGCTACTGGGGCAC
RS1467	dppA RP for RT-qPCR	AGATAACCGACGTTCAGCCC
RS1519	sugE FP for RT-qPCR	GGTCTGCTGGAAGTGGTATG
RS1520	sugE RP for RT-qPCR	TCCCCAATACGATTAACGCC
RS1546	pgaA FP for RT-qPCR	GGATCTCTTTTACAGCCACC
RS1547	pgaA RP for RT-qPCR	GTCATTTCAGCCTGAGGAAG
RS1578	yfjI FP for RT-qPCR	TACACACTTAACGAGCTGCC
RS1579	yfjI RP for RT-qPCR	TACCTTGTGTTGAAGCAGGC
RS1582	Ndh FP for RT-qPCR	GGCCTGCACACTAAAGATGG
RS1583	Ndh RP for RT-qPCR	AAAGTAACCATGCAGCGCAA
RS1593	alpA FP for RT-qPCR	ACTACGGTTACCAGCGGTTAT
RS1594	alpA RP for RT-qPCR	ATCTCGGCCTCAATCCATCC

RS1611	cspH FP for RT-qPCR	CGTATTTCCAGAGGGGGATAG
RS1612	cspH RP for RT-qPCR	CCGACAATACGTACGCTTGAG
RS1661	thiM FP for RT-qPCR	TTGATCCAGTAGCGGTGGG
RS1662	thiM RP for RT-qPCR	TTTCCCGTGCCAGTGTTTGT
RS1663	yhjV FP for RT-qPCR	GGTGGTGGTCAAAGTAGGGA
RS1664	yhjV RP for RT-qPCR	CGCTTCCCGTTTACGATAGG
RS1665	cadB FP for RT-qPCR	TATTCTGCTCTGCCTGTGGG
RS1666	cadB RP for RT-qPCR	AGCCGCCATTACAGAAGACG
RS1669	thiC FP for RT-qPCR	CCGCGAGTGGATTTTGCGTA
RS1670	thiC RP for RT-qPCR	CGCCCGCATGGATAGTGAA
RS1692	sanA RP for RT-qPCR	TTTAATCGCTGCTGGTGTCG
RS1693	sanA FP for RT-qPCR	GTGACGGTACGGCATAACAC
RS1694	sbp RP for RT-qPCR	CGAAGCAGATCCATGACTGG
RS1695	sbp FP for RT-qPCR	TGCCCGAACAAAATCCTGTG
RS1758	rsd RP for RT-qPCR	AAACGAAAAAGCCCTTGATG
RS1759	rsd FP for RT-qPCR	TTGAGTATTGGCTTCGAGTT
RS1760	rcnR FP for RT-qPCR	GCTCAAGAAAATGCTCGAC
RS1761	rcnR RP for RT-qPCR	CTTCACGTTTTAGCTCATCC
RS1762	cadC FP for RT-qPCR	ATCGCGTATTTTGCTCAATC
RS1763	cadC RP for RT-qPCR	AGTTGATTTTGTCATGCACC
RS1787	ydeT FP for RT-qPCR	GGTTTATGGCACTCTGCTGC
RS1788	ydeT RP for RT-qPCR	CCACTCCGACTGTAACCGAC
RS1791	yfhR FP for RT-qPCR	ACCCGAGCGTAATTTCAACG
RS1792	yfhR RP for RT-qPCR	CAATACTCTGCCCGAACAGC
RS1793	setC RP for RT-qPCR	TCGCTTATGAACTGGCAATGG
RS1818	rfaH FP for RT-qPCR	GCCATTGTTCCCCAACTACC
RS1819	rfaH RP for RT-qPCR	CACCCGGATAAGGGGTTGC
RS1824	waaZ FP for RT-qPCR	GGTCGTCTGGTTGGATTTTGC
RS1825	waaZ RP for RT-qPCR	CTTAATTCCGAGGGCATGGG
RS1830	rfaD FP for RT-qPCR	TTCCTGTACGCTTCTTCCGC
RS1831	rfaD RP for RT-qPCR	CTTCACGCGGTCCATAAACG
RS1834	dmsA FP for RT-qPCR	GTCGATAGCGCCATTCCAAC
RS1835	dmsA RP for RT-qPCR	TGCAGGCCGTCGTAATTGT
RS1836	ptsG FP for RT-qPCR	AATCGCCTCTAAACACCTGGC
RS1837	ptsG RP for RT-qPCR	GACAGCACAACGCCAGTAAAG
RS1838	osmY FP for RT-qPCR	TGACAACATCAAGAGCACCG
RS1839	osmY RP for RT-qPCR	ACCGAGCCTTCTTTAGCGTC
RS1840	topA FP for RT-qPCR	CCACATCTATCTCGCAACCGA
RS1841	topA RP for RT-qPCR	GCTGGGCATTAACACGATCA

RS1842	ygeQ FP for RT-qPCR	TGATGCTGTCTGGTTGAGGG
RS1843	ygeQ RP for RT-qPCR	TCCAGTCGTTTTGGGTTGCT
RS1848	gltF FP for RT-qPCR	GTACCCCAGTTGTTACGGGC
RS1849	gltF RP for RT-qPCR	AGCAATTAGCGTTGGTGCTG
RS1853	waaQ FP for RT-qPCR	CGAAGATTGCTGGAAGAAGATGC
RS1854	waaQ RP for RT-qPCR	GCAATCTCATCTACACAAGCGAG
RS1872	ykfG FP for RT-qPCR	GCGTAGCCTTCACCTCTACC
RS1873	ykfG RP for RT-qPCR	GAAGGATGGTTATGCGCGAG
RS1874	rpoC FP for RT-qPCR	CGTAAAGGTCTGGCGGATAC
RS1875	rpoC RP for RT-qPCR	TCAGCAGTTACACGACCCAG
RS1883	yafZ FP for RT-qPCR	AATCGCAGATCCTCTCCCC
RS1884	yafZ RP for RT-qPCR	ACAGTGCCCGGTTAAGTTTC
RS1887	selC FP for RT-qPCR	GCCTGTCGGTTCTTCTGTCC
RS1888	selC RP for RT-qPCR	GGTGTTCTGGGGCGAGTAAAA
RS1889	ydfU FP for RT-qPCR	GCGTGACCAGACATCGGAAT
RS1890	ydfU RP for RT-qPCR	TCCACCACCTGATTGCAGAC
RS1893	thrW FP for RT-qPCR	CGTAATGCGAAGGTCGTAGGT
RS1894	thrW RP for RT-qPCR	GCCAAGGATGTATAGTGAGCGA
RS1899	corA FP for RT-qPCR	TCGATGGTAGACGGTAACGC
RS1900	corA RP for RT-qPCR	ATCTTCCAGTTCCGCCAGAG
RS1905	yeeS FP for RT-qPCR	TTCGGTGATAAGCCGGTCTG
RS1906	yeeS RP for RT-qPCR	TCCGGGTGCTGTATCTGAAC
RS1909	yafQ FP for RT-qPCR	AGTTCCAGTTCTCTCAAATCG
RS1910	yafQ RP for RT-qPCR	GCACAAAAGCGTCATAAGG
RS1911	dinJ FP for RT-qPCR	ATAAATCATCGGCGTCTTTGGC
RS1912	dinJ RP for RT-qPCR	AAGAATCAGGCAGCGGACG
RS1943	yeiT FP for RT-qPCR	CGCGAAAATAATGCCCTCGG
RS1944	yeiT RP for RT-qPCR	GAGCGTTTTAGTACCGGGCT
RS1945	gntX FP for RT-qPCR	TTTGTTCGGTCTGTTCACGC
RS1946	gntX RP for RT-qPCR	TAAGCTGGTGGATAAGCGGA
RS1947	sodA FP for RT-qPCR	TCCCTTTTATCCCCGCGTTA
RS1948	sodA RP for RT-qPCR	TTCCGCCGTTTTACCACACA
RS1949	hcp FP for RT-qPCR	GCCAGCAGACAAGAAAACCG
RS1950	hcp RP for RT-qPCR	GAACCAAAGCGGGAAGCTG
RS1951	uvrB FP for RT-qPCR	AAATTACATACCTGCCCGCCC
RS1952	uvrB RP for RT-qPCR	AGGTTTTCCCTGAGCCAGTC
RS1953	ydhQ FP for RT-qPCR	TATCGTCTGGGGACGGATGG
RS1954	ydhQ RP for RT-qPCR	GGTGCGCGATCTTCCTCTAC
RS1955	topB FP for RT-qPCR	CGATAGCGTAAGCGAGTGAATC

RS1956	topB RP for RT-qPCR	AATACTTCTCGCTCCCAGGATG
RS1957	yiiS FP for RT-qPCR	TCGTAATCGACAGAGAGGCG
RS1958	yiiS RP for RT-qPCR	CGGCAAGCTCTTTCAGTTTGG
RS2119	tnaA FP for RT-qPCR	CCGCCAAGAAAGATGCGATG
RS2120	tnaA RP for RT-qPCR	CCGTCATACAGACCTACCGC
RS2123	ydeN FP for RT-qPCR	TGGGGTTATGCGGGTAATCG
RS2124	ydeN RP for RT-qPCR	TACCCGACAGCTCTTGATGC
RS2127	gatD FP for RT-qPCR	TGTTTGCATCGCACCGAAAG
RS2128	gatD RP for RT-qPCR	AGCCGATTACCGTTGGTCTG
RS2131	katE FP for RT-qPCR	TTGCTGAAAACGAACAGGCG
RS2132	katE RP for RT-qPCR	GTTCGGTTCGTAATTCGCCG
RS2137	pspB FP for RT-qPCR	AATCGTTCTGGTCGCAGTGA
RS2138	pspB RP for RT-qPCR	TCCAGCGCCTGAATACGTTC
RS2147	ydiL FP for RT-qPCR	AGACCGGTGACAGTGAAAGC
RS2148	ydiL RP for RT-qPCR	AATGCGGTTAAGTCGGGGAA
RS2149	puuD FP for RT-qPCR	CTGGCGGAACCGTCATTACT
RS2150	puuD RP for RT-qPCR	ATGCTCAGAAGATCACGCCC
RS2172	istR FP for RT-qPCR	GTGCCGAAATTGCGCGTTC
RS2173	istR RP for RT-qPCR	CGAGGTTTCGTCAGTCGCC
RS2174	ldrD FP for RT-qPCR	CACCACGGGGGCTAATCTTG
RS2175	ldrD RP for RT-qPCR	ATGACCGGAGCCGCTAAATC
RS2176	sokC FP for RT-qPCR	ATAGGAGGCCTCGGGTTGAT
RS2177	sokC RP for RT-qPCR	GGCTGTCTGTCTCAGGCATT
RS2013	FP for dmsA template for RNA synthesis	TAATACGACTCACTATAGGGAGAGCGTCCGTATATGAACGGACTGG
RS2014	RP for dmsA template for RNA synthesis	TAATGGCTCACTCAAGCTTGCTC
RS139	FP for LTR1 template for RNA synthesis	TTAATACGACTCACTATAGGGAGATCGAGAGGGACACGGGCG
RS341	RP for LTR1 template for RNA synthesis	GAATTGTGAGCGCTCACAATTCGGATCCTTAGATAACAATTGATTGAATG
RS 2035	thiC FP for RNA template synthesis	TAATACGACTCACTATAGGGAGACCGCGGAACCTGATCAGGC
RS 2036	thiC RP for RNA template synthesis	TCGTACTGCGGCTGTTCTTTGC
RS 2037	gfcAp FP for RNA template synthesis	TAATACGACTCACTATAGGGAGAGCTGCTTGTTTAAAGCTGACTGG
RS 2038	gfcAp RP for RNA template synthesis	CTATCCCCCTCTGGAAATACGAC
RS 2039	sodA FP for RNA template synthesis	TAATACGACTCACTATAGGGAGAACTGCTTACGCGGCATTAACAATC
RS 2040	sodA RP for RNA template synthesis	GGCTGTGGTTAGCGTGACC

RS 2041	sugE FP for RNA template synthesis	TAATACGACTCACTATAGGGAGATAGTTGAAATTCCCCTGCCACC
RS 2042	sugE RP for RNA template synthesis	TCCACACGGCATAAGCCGTC
RS 2043	yhjV FP for RNA template synthesis	TAATACGACTCACTATAGGGAGATTAGCCCCCGTGGTGTTGTC
RS 2044	yhjV RP for RNA template synthesis	GCAACAGATTCACTGGGGAGG
RS 2045	rsd FP for RNA template synthesis	TAATACGACTCACTATAGGGAGAGCCATTCGCCGATTTGTAGTGC
RS 2046	rsd RP for RNA template synthesis	AGTTTGTTACTTCCTCTGACGCG
RS 2047	gntX FP for RNA template synthesis	TAATACGACTCACTATAGGGAGAGTGACAGCGCACCAAATCCC
RS 2048	gntX RP for RNA template synthesis	CGTGAACAGACCGAACAAATCCC
RS 2049	ytfI FP for RNA template synthesis	TAATACGACTCACTATAGGGAGACTAAATTCAGATGGCAGAAACAGTG
RS 2050	ytfI RP for RNA template synthesis	GGCTATCATAATTCGAAATCCAATC
RS 2051	cysQ FP for RNA template synthesis	TAATACGACTCACTATAGGGAGACAGGGACATCCTTTTATCATCGG
RS 2052	cysQ RP for RNA template synthesis	TACCGGAGAATTGTCCGCTTTG
RS 2053	ydhQ FP for RNA template synthesis	TAATACGACTCACTATAGGGAGAGGGTGATTTTTATTCAGGATCGCA
RS 2054	ydhQ RP for RNA template synthesis	GTATTTTCGGCTCTTCCGCCAG
RS 2055	ycfJ FP for RNA template synthesis	TAATACGACTCACTATAGGGAGAGTGGCATAAACGCCTCATCCG
RS 2056	ycfJ RP for RNA template synthesis	CAACCTGAGCGTATTGCGGG
RS 2057	rac FP for RNA template synthesis	TAATACGACTCACTATAGGGAGAGCTTCACTGACATATTCTGCGAACA
RS 2058	rac RP for RNA template synthesis	CCTTACTGCATTAGGCACAGCC
